# Pex3 promotes formation of peroxisome-peroxisome and peroxisome-lipid droplet contact sites

**DOI:** 10.1038/s41598-025-07934-2

**Published:** 2025-07-08

**Authors:** Lucía Amado, Louis Percifull, Rico Franzkoch, Vico Flatemersch, Eleni Joana Brüggemann, Olympia Ekaterini Psathaki, Maya Schuldiner, Maria Bohnert, Margret H. Bülow, Ayelén González Montoro

**Affiliations:** 1 Cellular Communication Laboratory, Department of Biology/Chemistry, Osnabrück University, Barbarastrasse 13, 49076 Osnabrück, Germany; 2 Institute of Cell Dynamics and Imaging, University of Münster, Von-Esmarch-Str. 56, 48149 Münster, Germany; 3 Cells in Motion Interfaculty Centre (CiM), University of Münster, Münster, Germany; 4 iBiOs-Integrated Bioimaging Facility, University of Osnabrück, Osnabrück, Germany; 5 Center of Cellular Nanoanalytic Osnabrück (CellNanOs), Barbarastrasse 11, 49076 Osnabrück, Germany; 6 Division of Microbiology Department of Biology/Chemistry, Osnabrück University, Barbarastrasse 11, 49076 Osnabrück, Germany; 7 Group Membrane Contact Sites, CURE3D Research Lab, Clinic for Cardiovascular Surgery, University Hospital Düsseldorf, Moorenstraße 5, 40225 Düsseldorf, Germany; 8 Department of Molecular Genetics, The Weizmann Institute of Science, 7610001 Rehovot, Israel

**Keywords:** Peroxisomes, Proteomics

## Abstract

Peroxisomes are ubiquitous organelles that mediate central metabolic functions, such as fatty acid β-oxidation, as well as diverse tissue- and organism-specific processes. Membrane contact sites, regions of close apposition with other organelles for direct communication, are central to several aspects of their life cycle. Pex3 is a conserved multifunctional peroxisomal transmembrane protein that is involved in the insertion of peroxisomal membrane proteins, in pexophagy, and in the formation of membrane contact sites. Here, we show that high Pex3 levels in *Saccharomyces cerevisiae* induce the formation of peroxisome clusters surrounded by lipid droplets, mediated by peroxisome-peroxisome and peroxisome-lipid droplet contact sites. This clustering occurs independently of Pex3 partners in other processes Pex19, Inp1, and Atg36. The cytosolic domain of Pex3 binds peroxisomes, suggesting a direct role in homotypic contact site formation. Lipid droplet-peroxisome contact sites require the lipid droplet-localized triacylglycerol lipase Tgl4, which is enriched at this interface along with other lipases. Pex3 overexpression in *Drosophila melanogast*er similarly alters peroxisome and lipid droplet morphology and promotes contact site formation. Together, our results offer novel molecular insights into homotypic peroxisome contact sites and peroxisome-lipid droplet contact sites across species.

## Introduction

Peroxisomes are found in most eukaryotic cells and are the place of important metabolic reactions, including the oxidation of fatty acids, the synthesis of ether lipids, and the detoxification of hydrogen peroxide and glyoxylate. While some peroxisomal functions are tissue- or organism-specific, others are highly conserved, e.g. the oxidation of fatty acids, a process that is virtually ubiquitous^1^. Impaired peroxisome biogenesis as well as defects in peroxisomal metabolic pathways result in severe human diseases collectively known as peroxisomal disorders^1^.

Membrane contact sites are structurally defined regions of close organelle apposition without membrane fusion^2,3^. A central function of contact sites is the exchange of material among the compartments, including the transport of luminal material and of membrane lipids. Furthermore, the physical attachment of the organelle membranes can affect organelle fusion, fission, and positioning^4^. Membrane contact sites exist between virtually all pairs of organelles^5–7^ and coordinate multi-organelle processes^4,8^. Contact sites between peroxisomes and other organelles play important roles in different aspects of the peroxisome life cycle^9^. For example, in the yeast *Saccharomyces cerevisiae*, peroxisome contact sites with the endoplasmic reticulum affect peroxisome proliferation^10,11^ and the formation of peroxisome contact sites with the cell periphery determines the distribution of these organelles among mother and daughter cells^12^.

Pex3 is a peroxisomal membrane protein with multiple functions and interactors. It is involved in the targeting of peroxisomal membrane proteins, by acting as a docking factor for Pex19, a cytosolic receptor for peroxisomal membrane protein precursors. Another function of Pex3 is the recruitment of the pexophagy receptor Atg36. Both of these functions are conserved in the human Pex3 protein^13–19^. Pex3 has additional roles in contact site formation. It acts as a membrane anchor for Inp1, a tethering protein involved in the retention of peroxisomes in the mother cell during cell division, through its interaction with the cell cortex^12,20^. In the yeast *Hansenula polymorpha* extensive vacuole-peroxisome contact sites are formed during growth phases that require peroxisomal growth. Pex3 is enriched at these contact sites, and overexpression of the protein results in contact site expansion during growth in glucose, suggesting that Pex3 might be a tether^21^.

This prompted us to address the effects of Pex3 overexpression in *Saccharomyces cerevisiae*. We find that cells that overexpress Pex3 contain peroxisome clusters surrounded by lipid droplets, which are formed by peroxisome-peroxisome and peroxisome-lipid droplet contact sites. We further show that these contact sites are independent of all the known Pex3 interactors. Instead, efficient formation of peroxisome-lipid droplet contact sites requires the lipid droplet-localized TAG-lipase Tgl4 but not other lipid droplet lipases, even though several of them are enriched in the interface. Interestingly, similar effects of Pex3 overexpression on peroxisome morphology and extended contact with the lipid droplets were also observed in *Drosophila melanogaster*, showing that these aspects of Pex3 function are conserved to metazoa. Altogether, our findings expand our understanding of peroxisomal contact sites, and of the multi-functional protein Pex3.

## Results

### Overexpression of Pex3 causes a change in the morphology of peroxisomes and lipid droplets

**Fig. 1:**
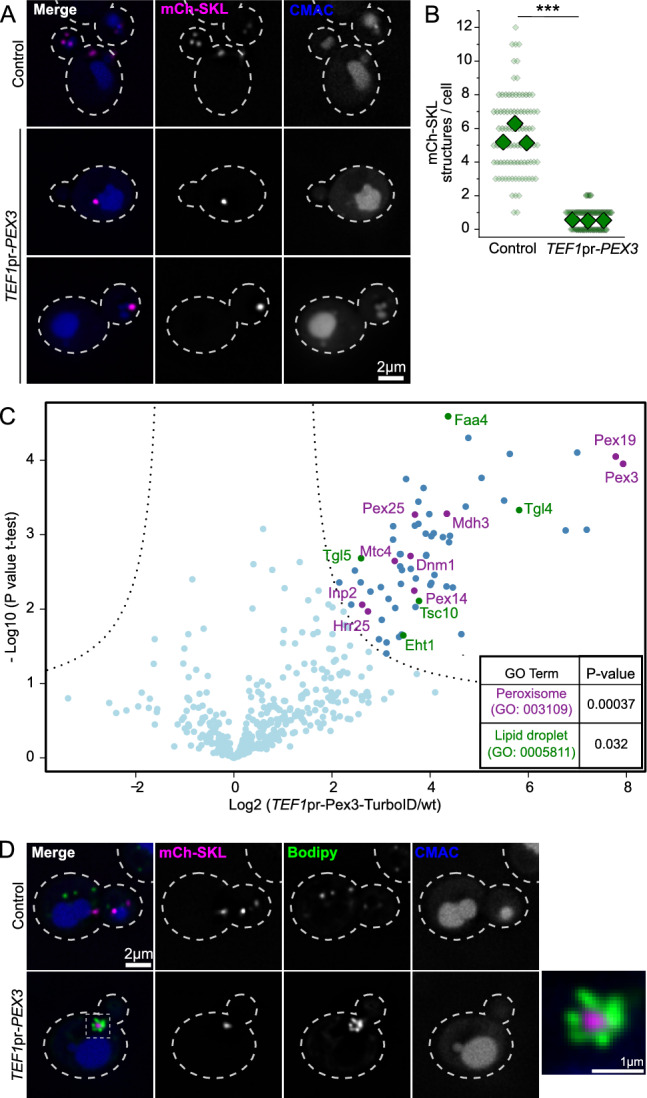
Overexpression of Pex3 causes the formation of a single peroxisomal structure surrounded by lipid droplets. (**A–B**) Overexpression of Pex3 produces the collapse of all peroxisomal signal into one structure. Panel A shows representative pictures of a strain expressing mCherry-SKL construct to visualize the lumen of the peroxisomes, either with Pex3 at endogenous levels (Control) or overexpressed (*TEF1*pr-*PEX3*) and the vacuolar lumen stained with CMAC. Cell outlines are shown as white dashed lines. Scale bar: 2 μm. Panel B shows the quantification of the amount of peroxisomal structures per cell. Three independent experiments were performed and 30 cells were analyzed for each experiment and condition. Small diamonds correspond to individual cells, bigger diamonds correspond to the average of independent experiments. The different strains were compared using an unpaired two-tailed Student’s t-test. *** P < 0.001. (**C**) Turbo ID of overexpressed Pex3-TurboID enriches peroxisomal and lipid droplets proteins. Volcano plot showing relative protein intensity in a pull-down of biotinylated proteins between a strain overexpressing Pex3 tagged c-terminally with the TurboID protein (*TEF1*pr-*PEX3*-TID) and a wild type control strain (wt). Peroxisomal proteins are marked in magenta and lipid droplets proteins in green. GO term enrichment analysis of the group of proteins significantly enriched in the Pex3-TID pull-down showed an enrichment of the GO Terms “peroxisomes” and “lipid droplets”. (**D**) The formed peroxisomal structure is surrounded by lipid droplets. Representative pictures of a strain expressing mCherry-SKL construct to visualize the lumen of the peroxisomes, either with Pex3 at endogenous levels (Control) or overexpressed (*TEF1*pr-*PEX3*), the vacuolar lumen stained with CMAC and lipid droplets stained with Bodipy. Scale bar: 2 μm. The zoomed in region shows a peroxisomal structure surrounded by lipid droplets with a scale bar of 1 μm. Cell outlines are shown as white dashed lines.

In *Hansenula polymorpha*, Pex3 was observed to be enriched in membrane contact sites between peroxisomes and the vacuole, and overexpression of this protein results in an extension of these contact sites^21^. Thus, we decided to test the phenotype of Pex3 overexpression in *Saccharomyces cerevisiae.* Overexpression from the strong constitutive *TEF1* promoter resulted in a morphological change in peroxisomes marked by mCherry directed to the peroxisomal lumen by a PTS1 signal consisting of a serine-lysine-leucine sequence (mCh-SKL). While control cells show on average 5.5 mCh-SKL positive structures, cells overexpressing Pex3 contain mainly one structure (Fig. 1A and B). This structure was found in close proximity to the vacuole in 55% of cells. We confirmed these observations by using other peroxisomal markers, Pex3 itself and Pex14, obtaining similar results (Supplemental Fig. 1A and B). We analyzed the distribution of this peroxisomal structure among mother and daughter cells and found that it can be found in both compartments, with some preference for the bud (Supplemental Fig. 1C).

To characterize this structure we sought to describe its molecular microenvironment by proximity biotinylation^22^, by tagging Pex3 with TurboID^23^ at its C-terminus, which faces the cytosol. Cells expressing Pex3-TurboID under the *TEF1* promoter were incubated with biotin for 3 h and the biotinylated proteins were isolated by affinity chromatography using a streptavidin matrix. The bait protein Pex3 was among the most highly enriched proteins, as was its known interactor Pex19 (Fig. 1C). Gene Ontology (GO) Term enrichment analysis showed the GO term “Peroxisome” as the most represented annotation among our enriched proteins, as expected. Interestingly, the GO term “Lipid droplet” was also significantly enriched (*P* value = 0.032, Fig. 1C).

This prompted us to address the subcellular localization of lipid droplets when Pex3 is overexpressed by fluorescence microscopy. This analysis revealed that under these conditions, lipid droplets are strongly recruited to the peroxisomal structure (Fig. 1D). We observed and quantified the presence of LDs in close proximity of peroxisomal structures in 90 cells from three independent experiments. Upon Pex3 overexpression, 95% of the peroxisomal structures were in close proximity to or completely surrounded by lipid droplets, whereas the remaining 5% of peroxisomal structures did not present any lipid droplet in their vicinity (Fig. 1D). The size of lipid droplets is also slightly increased under these conditions (Supplemental Fig. 1D). We conclude that overexpression of Pex3 causes a change in the morphology of peroxisomes and lipid droplets, resulting in the observation of a single peroxisomal structure per cell, which is in close proximity to lipid droplets.

### Pex3 overexpression induces a cluster of peroxisomes surrounded by lipid droplets, which includes peroxisome-peroxisome and peroxisome-lipid droplet contact sites

**Fig. 2:**
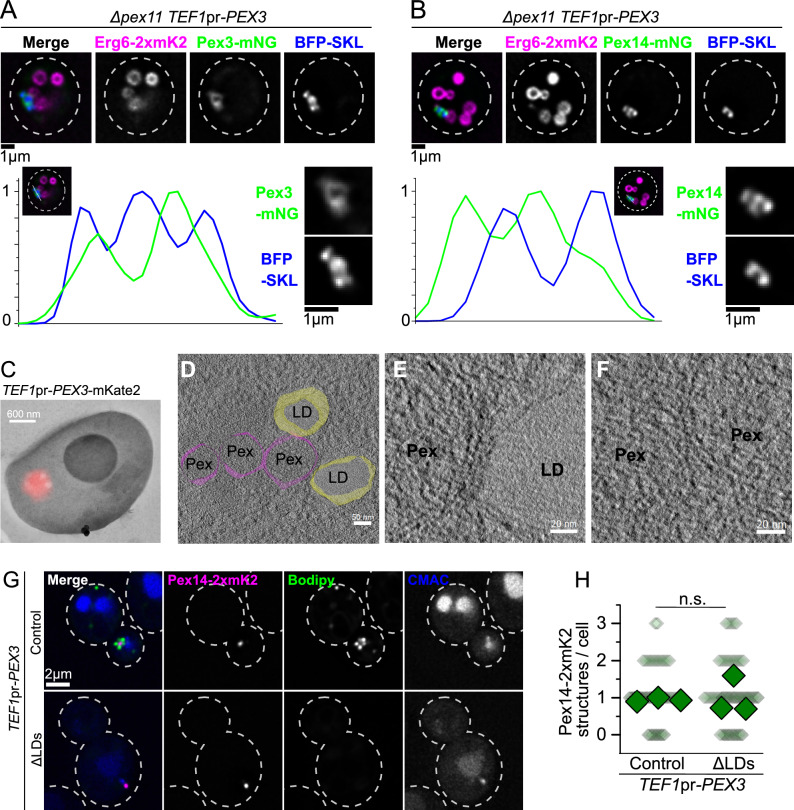
Overexpression of Pex3 induces an accumulation of peroxisomes surrounded by LDs that contains Pex-Pex and Pex-LD contact sites. (**A–B)** Enlarged peroxisomes and LDs reveal that the structures contain several maxima for peroxisome lumen signal, with peroxisomal membrane between them. Representative pictures of a strain with overexpressed Pex3 (*TEF1*pr-*PEX3*), expressing the BFP-SKL construct to visualize the lumen of the peroxisomes, Erg6-2xmKate2 marking the lipid droplet monolayer, and either Pex3 (A) or Pex14 (B) tagged with mNeonGreen as markers of the peroxisomal membrane. To produce enlarged peroxisomes and lipid droplets, the cells contain a deletion of *PEX11*, and were grown with oleate as the sole carbon source for 20hs. Cell outlines are shown as white dashed lines. Scale bars: 1 μm. Each graph shows the signal of BFP-SKL and the corresponding peroxisomal membrane protein over a line across the structure, depicted in the merged image. (**C–F**) On-section CLEM tomography confirms that the structure involves Pex-Pex and Pex-LD contact sites. Panel C shows a representative image of on-section CLEM done on a strain with overexpressed Pex3 (*TEF1*pr-*PEX3*) and tagged with 2xmKate2. Scale bar: 600 nm. Panel D shows a tomography image of the same section overlayed with the 3D model recreated from the images, showing peroxisomes in magenta and lipid droplets in yellow. Scale bar: 50 nm. Panels E and F show zoomed in regions of the tomogram as examples of the peroxisome-lipid droplet (E) and peroxisome-peroxisome (F) contact sites. Scale bars: 20 nm. (**G–H**) LDs are not necessary for the formation of the cluster of peroxisomes. Panel G shows representative pictures of strains with overexpressed Pex3 (*TEF1*pr-*PEX3*) in control cells and in strains that cannot produce lipid droplets (ΔLDs), expressing Pex14-2xmKate2 to visualize the peroxisomes, lipid droplets were stained with Bodipy and the vacuolar lumen was stained with CMAC. Cell outlines are shown as white dashed lines. Scale bar: 2 μm. Panel H shows the quantification of the amount of peroxisomal structures per cell. Three independent experiments were performed and 30 cells were analyzed for each experiment and condition. Small diamonds correspond to individual cells, bigger diamonds correspond to the average of independent experiments. The different strains were compared using an unpaired two-tailed Student’s t-test. n.s., not significant.

To understand the characteristics of the structure formed by lipid droplets and peroxisomes upon Pex3 overexpression, we sought to enlarge the structure to gain spatial resolution. This was achieved by deleting *PEX11*, which results in enlarged peroxisomes^24,25^. Additionally, we cultured the cells in the presence of oleate, which resulted in enlarged lipid droplets. These conditions allowed sufficient spatial resolution to distinguish the peroxisomal matrix from the membrane, as peroxisomal membrane proteins (Pex13 and Pex3) were observed to surround BFP directed to the peroxisomal lumen by a C-terminal PTS1 signal (BFP-SKL) (Fig. 2A and B, Supplemental Fig. 1E–G). Similar to what we observed before, Pex3 overexpression caused clustering of the peroxisomal signal. The increased resolution provided by these conditions allowed us to observe that these clusters contain multiple individual maxima of the BFP-SKL signal (Supplemental Fig. 1E–G). Figure 2A and B show examples of these clusters, as well as line profiles across them, illustrating that the peroxisomal membrane proteins show peaks between the individual maxima of the BFP-SKL signal (Fig. 2A and B). This indicates that the peroxisomal structure formed upon Pex3 overexpression corresponds to a cluster of multiple peroxisomes. The lipid droplets marked by Erg6-2xmKate2 were observed to surround the cluster of peroxisomes.

Given the close and specific proximity observed between these organelles upon overexpression of Pex3, we reasoned that such an organelle cluster would likely be formed by peroxisome-peroxisome and peroxisome-lipid droplet contact sites. To test this, we performed on-section CLEM tomography of cells overexpressing Pex3-mKate2, using the mKate2 signal to locate the structures. This approach revealed clusters of peroxisomes surrounded by lipid droplets that included peroxisome-peroxisome and peroxisome-lipid droplet contact sites (Fig. 2C–D). Figure 2C shows a on-section correlative fluorescence microscopy and TEM image from a cell overexpressing Pex3-mKate2, used to identify the relevant region for TEM tomography. Figure 2D shows imaging of this region by TEM tomography, overlayed with a 3D model reconstructing the observed organelles. Figure 2E and F and Supplemental videos 1 and 2 show example regions of the tomogram, containing peroxisome-lipid droplet and peroxisome-peroxisome contact sites, respectively.

We asked if the formation of peroxisome-peroxisome contact sites and peroxisome-lipid droplet contact sites occurred independently, or whether the clustering of the two types of organelles was interlinked. To test this, we overexpressed Pex3 in a strain that is devoid of lipid droplets because it lacks the synthases for triglycerides and sterol esters, namely Dga1, Lro1, Are1 and Are2 (from here on termed ΔLDs)^26^. Overexpression of Pex3 caused the accumulation of peroxisomal signal into a single structure irrespective of the absence of lipid droplets, showing that lipid droplets are not required for the formation of the peroxisome-peroxisome contact sites (Fig. 2G and H).

### The cytosolic domain of Pex3 interacts with peroxisomes

**Fig. 3:**
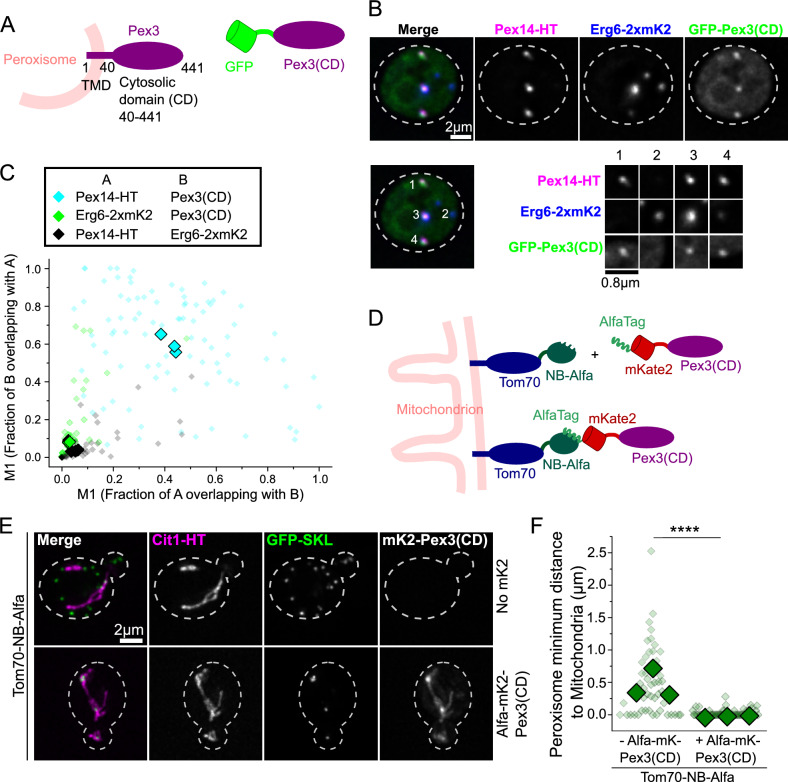
The cytosolic domain of Pex3 binds peroxisomes and is able to tether them to another organelle. (**A–C**) The cytosolic domain of Pex3 binds to peroxisomes. Panel A shows a diagram of the GFP-Pex3(CD) construct. This construct contains the cytosolic domain of Pex3 (aa40-441) fused to GFP tag at the N-terminus instead of the transmembrane domain as in the full length Pex3. Panel B shows representative pictures of the colocalization experiment of GFP-Pex3(CD) with peroxisomes (Pex14-HaloTag) and lipid droplets (Erg6-2xmKate2). Cell outlines are shown as white dashed lines. Scale bar: 2 μm and 0.8 μm. Panel C shows the co-localization analysis of the experiment in B using Mander´s coefficients M1 and M2 for the overlap of: Pex14-HaloTag and GFP-Pex3(CD) (cyan diamonds), Erg6-2xmKate2 and GFP-Pex3(CD) (green diamonds) or Pex14-HaloTag and Erg6-2xmKate2 (black diamonds). Three independent experiments were performed and 30 cells were analyzed for each experiment. Each small diamond represents a single cell, and the bigger ones represent the average of each of three independent experiments. (**D**) Diagram of the strategy used to recruit the cytosolic domain of Pex3 to mitochondria artificially. The outer mitochondrial membrane protein Tom70 was tagged in the C-terminus with a Nanobody that recognizes the AlfaTag (Tom70-NB-Alfa). The cytosolic domain of Pex3 was tagged with an AlfaTag and an mKate2 fluorescent protein in the N-terminus (AlfaTag-mKate2-Pex3(CD) construct). This causes the recruitment of AlfaTag-mKate2-Pex3(CD) to mitochondria. (**E–F**) Targeting the cytosolic domain of Pex3 to mitochondria tethers peroxisomes to this organelle. Panel E shows representative images of the localization of peroxisomes (GFP-SKL) and mitochondria (Cit1-HaloTag) in the presence or absence of AlfaTag-Pex3(CD), with Tom70 fused to Alfa Nanobody in the background. A maximum intensity projection of the Z-stacks is shown for each image. Cell outlines are shown as white dashed lines. Scale bar: 2 μm. Panel F shows the measurements of distances between peroxisomes (GFP-SKL) and mitochondria (Cit1-HaloTag) in the presence or absence of the AlfaTag-Pex3(CD) construct as described. Three independent experiments were performed and 30 cells were analyzed for each experiment. Each small diamond represents a single peroxisome, and the bigger ones represent the average of each of three independent experiments. The distribution of distances to mitochondria between the two strains was compared using a Kolmogorov-Smirnoff test, **** P < 0.0001. Comparison of the means of each experiment with a two-tailed unpaired Student´s t-test results in a P value < 0.05.

Pex3 is anchored to the peroxisomal membrane by a single transmembrane domain at its N-terminus, which is sufficient to cause targeting to the peroxisomes, and contains a globular cytosolic C-terminal domain^27–29^ (Fig. 3A). To address a possible direct involvement of Pex3 in contact site formation, we expressed the cytosolic domain (CD – amino acids 40–441) fused to GFP at its N-terminus and lacking the transmembrane region (Fig. 3A). We observed that this construct localized at peroxisomes, marked by Pex14-HaloTag (Fig. 3B). The construct did not decorate lipid droplets marked by Erg6-2xmKate2, and was only observed enriched in these structures when peroxisomal signal was also present (Fig. 3B, see zoomed in organelles). Consistently, calculation of the Mander´s coefficients M1 and M2 for GFP-Pex3(CD) with Pex14-HaloTag showed high values, while the coefficients of overlap with the Erg6-2xmKate2 signal were much lower and comparable to the ones observed between Pex14-HaloTag and Erg6-2xmKate2 (Fig. 3C).

To test if the interaction of the cytosolic domain of Pex3 with peroxisomes is strong enough to enable organelle tethering, we artificially directed this domain to mitochondria. This was achieved by tagging it with the fluorescent protein mKate2 and an N-terminal Alfa tag^30^, In addition, the mitochondrial outer membrane receptor Tom70 was tagged with a nanobody that recognizes the Alfa tag^30^, so it should direct the Pex3(CD) to mitochondria (Fig. 3D). Indeed, Alfa-mKate2-Pex3(CD) decorates the mitochondrial network under these conditions, as confirmed by co-localization with the mitochondrial marker Cit1-HaloTag (Fig. 3E). We quantified the minimum distance of each individual peroxisome to the mitochondrial network, for cells either expressing or not expressing the Alfa-mKate2-Pex3(CD) construct. The expression of the construct caused a strong shift to smaller distances (Fig. 3F). Figure 3E shows a representative image of the experiment, and additional examples are shown in Supplemental Fig. 2. This data indicates that the interaction of Pex3(CD) with peroxisomes is strong enough to induce organelle tethering. Based on these results we propose that the homotypic peroxisomal contact sites are formed by Pex3 being anchored to the peroxisomal membrane via its transmembrane domain, and additionally interacting with other peroxisomes through its cytosolic domain. In contrast, the peroxisome-lipid droplet contact site is likely not directly mediated by Pex3, and only indirectly induced by its overexpression.

### Known interactors of Pex3 are not involved in forming the peroxisome-lipid droplet cluster

**Fig. 4:**
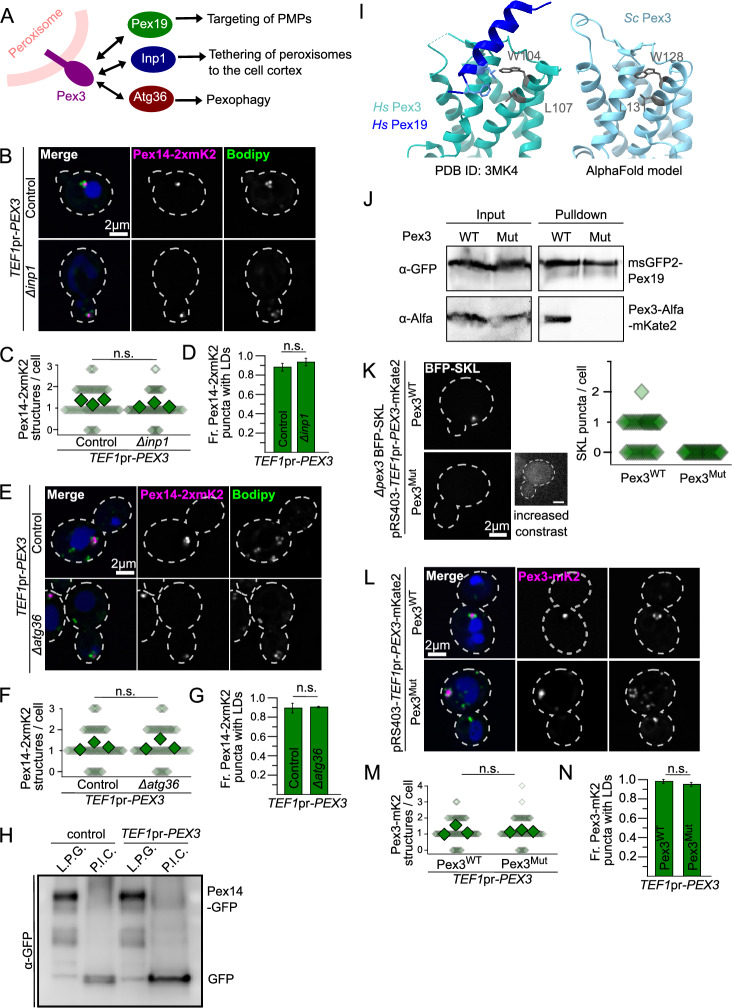
Formation of the structure is independent of known interactors of Pex3. (**A**) Diagram of Pex3 known interactors and their functions. (**B, E**) Representative images of strains overexpressing Pex3 (*TEF1*pr-*PEX3*) in control cells and strains lacking Inp1 (B) or Atg36 (E). All strains express Pex14 fused to 2xmKate2 to visualize the peroxisomes, lipid droplets were stained with Bodipy and the vacuolar lumen was stained with CMAC. Cell outlines are shown as white dashed lines. Scale bars: 2 μm. (**C, F**) Quantification of the number of peroxisomal structures per cell in the microscopy experiments described before. Small diamonds correspond to individual cells, bigger diamonds correspond to the average of independent experiments. Three independent experiments were performed and 30 cells were analyzed for each experiment and condition. The different strains were compared using an unpaired two-tailed Student’s t-test. n.s., not significant. (**D, G**) Quantification of the fraction of peroxisomal structures with accumulations of lipid droplets next to them in the microscopy experiments described above. Three independent experiments were performed and 30 cells were analyzed for each experiment and condition. The different strains were compared using an unpaired two-tailed Student’s t-test. n.s., not significant. (**H**) Pex3 overexpression does not induce pexophagy. Whole cell lysates of strains expressing Pex14-GFP with or without Pex3 overexpression were analyzed by Western blot. The cells were either grown in media containing glucose to logarithmic phase (L.P.G) or grown in media containing oleate and shifted to nitrogen starvation medium to induce pexophagy (P.I.C). The presence of a free GFP band is indicative of vacuolar degradation of peroxisomes. The whole Western blot membrane as well as a loading control is shown in Supplemental Fig. 3 A. (**I**) The amino acids involved in hsPex3 interaction with hsPex19 are conserved in yeast. To the left, the structure obtained for *Homo sapiens* Pex3 (cyan) interacting with a peptide of hsPex19 (dark blue)^17^. Amino acids W104 and L107 of hsPex3 are involved in the interaction with hsPex19. To the right, the structure predicted by AlphaFold for *Saccharomyces cerevisiae* Pex3 (light blue) shows that it contains a structurally conserved W and L in the same positions (W128 and L131 in scPex3). (**J**) Pex3(W128K, L131K) cannot interact with Pex19. Affinity purification of msGFP2-Pex19 co-purifies Pex3-mKate2-AlfaTag but not Pex3(W128K, L131K)-mKate2-AlfaTag. The complete Western blot membranes are shown in Supplemental Fig. 3 B and C. (**K**) Pex3(W128K, L131K) does not support BFP-SKL import into peroxisomes. In a strain that expresses BFP-SKL, endogenous Pex3 was deleted and either Pex3wt or Pex3(W128K, L128K) were re-introduced in a plasmid. Quantification of the number of BFP-SKL puncta per cell is shown to the right. (**L–N**) Overexpression of Pex3(W128K, L131K), which cannot interact with Pex19, still causes aggregation of peroxisomes and recruitment of lipid droplets. Panel K shows representative images of strains overexpressing Pex3 from a plasmid (*TEF1*pr-*PEX3*-mKate2) containing either Pex3 wt or Pex3 mutant. Lipid droplets were stained with Bodipy and the vacuolar lumen was stained with CMAC. The strain contains Pex3 wt in the background, expressed from its genomic locus, in order to have normal peroxisomes. Cell outlines are shown as white dashed lines. Scale bars: 2 μm. Panel L shows the quantification of the number of peroxisomal structures per cell in the microscopy experiments described before. Small diamonds correspond to individual cells, bigger diamonds correspond to the average of independent experiments. Panel M shows the quantification of the proportion of peroxisomal structures with accumulations of lipid droplets next to them in the microscopy experiments described above. Three independent experiments were performed and 30 cells were analyzed for each experiment and condition. The different strains were compared using an unpaired two-tailed Student’s t-test. n.s., not significant.

Pex3 is a multifunctional protein involved in different processes related to the life-cycle of peroxisomes, including the targeting of peroxisomal membrane proteins, pexophagy, and targeting of peroxisomes to the cortex, which strongly influences peroxisome inheritance^12–17,19,29^. These functions are mediated by the direct interaction of Pex3 with different binding partners (Fig. 4A). Targeting of peroxisomal membrane proteins to the peroxisomal membrane involves its interaction with the cytosolic receptor Pex19^14,17^ while its role in autophagy is mediated by its interaction with the pexophagy receptor Atg36^15^. Finally, the tethering of peroxisomes to the cell cortex is mediated by Pex3 interacting with Inp1^12,20^ (Fig. 4A). Next, we tested if any of the known interactors of Pex3 is involved in the formation of the peroxisome-lipid droplet cluster. To do this, we overexpressed Pex3 in strains lacking either *ATG36* or *INP1.* The representative microscopy images and quantifications shown in Fig. 4B–G show that the phenotype caused by Pex3 overexpression in these backgrounds does not differ from the control cells, indicating that neither Inp1 nor Atg36 are required for the formation of this structure.

The fact that Atg36 is not required for the formation of this structure suggests that it is not an intermediary in the process of pexophagy. However, we decided to test if pexophagy is induced by Pex3 overexpression, since this has been reported in mammalian cell lines^19^ In yeast, GFP is resistant to vacuolar degradation, and thus the generation of a free GFP band has been used as a readout for vacuolar degradation of different proteins and organelles, including peroxisomes^31–33^. Thus, we compared the appearance of a free GFP band in cells overexpressing Pex3 with cells grown under known pexophagy-inducing conditions, namely growth in oleate medium followed by nitrogen starvation^33^. Figure 4H and Supplemental Fig. 3A, B and C show that in cells expressing Pex14-GFP, a free GFP band appears under pexophagy-inducing conditions (P.I.C), indicating vacuolar degradation of peroxisomes. In contrast, the lack of a free GFP band in the strain overexpressing Pex3 when grown to logarithmic phase in glucose (L.P.G.) shows that overexpression of this protein alone does not induce pexophagy. Interestingly, pexophagy can still be induced in this strain, suggesting that it is also not blocked by the formation of the peroxisomal clusters.

To test the requirement of *PEX19*, we could not delete this gene, as this results in the absence of functional peroxisomes^34^. We thus used an alternative strategy, by addressing whether overexpression of a mutant version of Pex3 that does not interact with Pex19 still promotes the formation of the peroxisome-lipid droplet cluster. The crystal structure of human Pex3 in complex with a fragment of human Pex19 has previously been solved. It was found that the interaction with Pex19 is mediated by a region centered around HsPex3-Trp104, which also includes Leu107^16,39^. Alignment of the AlphaFold-generated structure prediction of ScPex3 with the structure of HsPex3 indicated that this region is highly conserved and that the equivalent residues in ScPex3 are Trp128 and Leu131 (Fig. 4I). We thus introduced the mutations Trp128Lys and Leu131Lys in ScPex3 (Pex3^Mut^) and tested the ability of this mutant to interact with Pex19, support peroxisome biogenesis, and form the peroxisome-lipid droplet structures upon overexpression. Unlike wtPex3, Pex3^Mut^ was not co-purified with msGFP2-Pex19, indicating that the mutations disrupt this interaction (Fig. 4J and Supplemental Fig. 3 D, E and F). The Pex19-Pex3 interaction has been reported to be required to import PTS1 containing peroxisomal matrix proteins^14^. Consistently, we observed that expression of Pex3^Mut^ in a strain lacking endogenous Pex3 does not rescue the import of BFP-SKL into peroxisomes, confirming that this mutation disrupts the interaction (Fig. 4K). Figure 4L–N contain representative microscopy images and the corresponding quantification showing that overexpression of this mutant in a background containing endogenous levels of wtPex3 to have functional peroxisomes, causes the same morphological phenotype as the overexpression of wtPex3. Thus, the interaction with Pex19 is not required for Pex3 to induce clustering of peroxisomes and lipid droplets.

### The triacylglycerol lipase Tgl4 is involved in the formation of the peroxisome-lipid droplet contact sites

We sought to identify additional molecular players involved in the formation of contact sites upon Pex3 overexpression. Since many contact site tether proteins are involved in the formation of more than one contact site^35–41^, we tested the involvement of known tether proteins of lipid droplets or peroxisomes with other organelles. Deletion of the peroxisomal-mitochondrial tethers Pex34 or Fzo1^1^ did not affect formation of Pex3-dependent peroxisome and lipid droplet clusters (Supplemental Fig. 4A), indicating that they are not required for formation of the contact sites. We also tested the involvement of the the splicing-generated pair of proteins Ldo16-Ldo45, which tether lipid droplets to the vacuole^42,43^. Deletion of these genes did not affect the clustering of peroxisomes or lipid droplets, nor the proximity of the clustered structure to the vacuole (Supplemental Fig. 4B). Finally, it was recently shown that the human protein M1 Spastin tethers lipid droplets to peroxisomes^44^. We deleted the yeast homolog Sap1, but observed no alteration of the Pex3-dependent phenotype (Supplemental Fig. 4C).

Peroxisomes and lipid droplets are also linked by the role of the endoplasmic reticulum during their biogenesis. Both lipid droplets and pre-peroxisomal vesicles bud from a subdomain of the endoplasmic reticulum marked by the reticulon-like protein Pex30^45^. It was reported that the same domain can bind simultaneously to a lipid droplet and a peroxisome, causing a close association between them^46^. Deletion of *PEX30* did not affect the formation of the peroxisomal and lipid droplet clusters (Supplemental Fig. 4D), indicating that this protein is not directly involved in forming them.

**Fig. 5:**
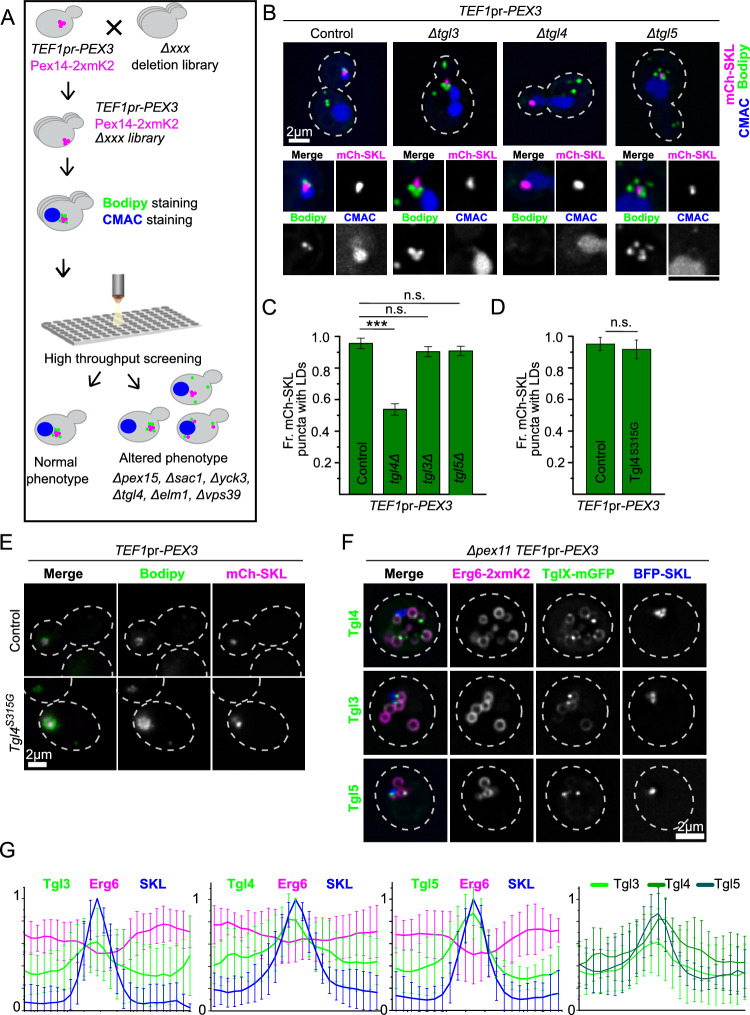
A genome-wide microscopy-based screen identifies that deletion of Tgl4 interferes with the targeting of LDs to the peroxisomal structure. (**A**) A microscopy-based screen for deletions that disrupt the Pex3 overexpression phenotype. Establishment of a deletion library with the overexpression of Pex3 (*TEF1*pr-*PEX3*) by SGA and subsequent automated high-content microscopy-based screen. Deletion of *TGL4* was identified to disrupt the phenotype. (**B–C**) Deletion of TGL4 disrupts Per-LD CSs, and this effect is specific for this lipase. Panel B shows representative images of strains overexpressing Pex3 (*TEF1*pr-*PEX3*) in control cells and strains lacking either Tgl3, Tgl4 or Tgl5. All strains express mCherry-SKL construct to visualize the lumen of the peroxisomes, lipid droplets were stained with Bodipy and the vacuolar lumen was stained with CMAC. Cell outlines are shown as white dashed lines. Scale bars: 2 μm. Panel C shows the quantification of the proportion of peroxisomal structures with accumulations of lipid droplets next to them in the microscopy experiments described above. Three independent experiments were performed and 30 cells were analyzed for each experiment and condition. The different strains were compared by ANOVA and a post-hoc Tukey test. n.s., not significant, *** P < 0.001. (**D–E**) The role of Tgl4 in establishing this contact site is independent from its lipase activity. Panel E shows representative images of strains overexpressing Pex3 (*TEF1*pr-*PEX3*) in control cells and in a strain with Tgl4 (S315G) punctual mutant in the background. Strains express BFP-SKL construct to visualize the lumen of the peroxisomes and Erg6 fused to 2xmKate2 to visualize the lipid droplets. Cell outlines are shown as white dashed lines. Scale bars: 2 μm**.** Panel D shows the quantification of the proportion of peroxisomal structures with accumulations of lipid droplets next to them. Three independent experiments were performed and 30 cells were analyzed for each experiment and condition. The two different strains were compared using an unpaired two-tailed Student’s t-test. n.s., not significant. (**F–G**) Enlarged peroxisomes and LDs reveal that Tgl3, Tlg4 and Tgl5 are enriched at the interfaces at Per-LD CSs to different degrees. Representative pictures of a strain with overexpressed Pex3 (*TEF1*pr-*PEX3*), expressing the BFP-SKL construct to visualize the lumen of the peroxisomes, Erg6-2xmKate2 marking the lipid droplet monolayer, and each of the Tgl proteins tagged with mGFP. To produce enlarged peroxisomes and lipid droplets, the cells contain a deletion of *PEX11*, and were grown with oleate as the sole carbon source for 20hs. Cell outlines are shown as white dashed lines. Scale bars: 2 μm. The panels in G, show average line profiles of the BFP-SKL, Erg6-2xmKate and TglX-GFP signal around lipid droplets. The lines started at the opposite end of the peroxisome contact site, and aligned using the maxima of the BFP-SKL signal. 10 Cells were averaged for each strain. The final panel shows an overlay of the average line profiles of the different Tgl lipases, to compare the relative intensities.

We next performed a genome-wide microscopy-based screen, to identify factors affecting the formation of this cluster. We crossed a strain carrying *TEF1pr*-Pex3 and the peroxisomal marker Pex14-mKate2 with a genome-wide collection of deletion mutants of non-essential genes^47^ and hypomorphic DAmP allele mutants of essential genes^48^ using an automated mating and sporulation procedure^49,50^. The resulting mutant collection contains *TEF1pr*-Pex3, Pex14-2xmKate2 and each gene deleted or depleted (Fig. 5A). We analyzed this collection by automated microscopy after labeling cells with Bodipy and CMAC, to stain lipid droplets and vacuoles respectively. The resulting images were analyzed manually searching for strains in which the phenotype was disrupted. We found a single hit in which the phenotype was significantly disrupted, which carried the deletions of the gene encoding for the lipase Tgl4^51^.

This strain was manually re-constructed to confirm that the disruption of the phenotype did not depend on the genetic background used for the screen and re-analyzed by microscopy (Fig. 5B). In cells that overexpressed Pex3, 95% of the peroxisomal structures were proximal to lipid droplets, while this number dropped to 51% in cells that in addition lacked Tgl4 (Fig. 5C). Re-insertion of the *TGL4* ORF with its endogenous promoter in a plasmid fully recovered the interaction between peroxisomes and lipid droplets, indicating that the effect is specific to the lack of the gene, and not a secondary effect of the genomic modification (Supplemental Fig. 5A and B).

We tested the specificity of the phenotype by deleting other lipid droplet-localized TAG lipases. Neither deletion of the genes encoding for the lipases homologous to Tgl4, namely Tgl3 and Tgl5^52,53^ (Fig. 5B and C) nor other ones, Tgl1, Ldh1 or Yeh1^54–56^ (Supplemental Fig. 5C and D) caused a disruption of the phenotype. The homologous lipases also did not cause a further disruption when combined with the deletion of *TGL4* (Supplemental Fig. 5E and F). This suggests that it is the physical presence of the protein Tgl4 that affects the formation of the contact site and not its activity as a lipase. To test this hypothesis, we assessed the effect of mutation S315G, which disrupts its active site^57^, and observed no reduction in the association of lipid droplets to the peroxisomal cluster (Fig. 5D and E).

In addition, we analyzed the localization of Tgl3, 4, and 5 on the lipid droplet surface upon induction of these contact sites. Again, we used the strain lacking *PEX11* and grew the cells in the presence of oleate to increase the size of peroxisomes and lipid droplets and gain spatial resolution. We observed that all three lipases were enriched in the region of the lipid droplet that was in contact with the peroxisomes in some cells, as can be appreciated in the example images and line profiles (Fig. 5F and G). These enrichments, however, were not equally frequent for all lipases: Tgl4 and Tgl5 were enriched more frequently than Tgl3. This can be observed by the resulting peaks formed by averaging many cells in the line profiles (Fig. 5G).

To explore the requirements for the formation of Tgl4 foci at lipid droplet-peroxisome interfaces, we performed a microscopy-based screen (Supplemental Fig. 6A). A *PEX3* overexpression allele, genes for lipid droplet and peroxisome visualization (Erg6-mCh and BFP-SKL), and Tgl4-GFP were introduced into the genome-wide deletion and DAmP libraries^47,48^ by an automated mating approach^49,50^. Cells were cultured in the presence of oleate to expand lipid droplets and analyzed by automated microscopy. We identified a total of 86 mutants in which the accumulation of Tgl4-GFP foci at lipid droplet-peroxisome interfaces was fully or partially blocked (Supplemental Table 5, example microscopy images in Supplemental Fig. 6B). We analyzed the common functions among the genes identified by the screen (Supplemental Fig. 6C). The biggest group of genes was related to energy metabolism. This is also evidenced by the enrichment of the GO Terms “mitochondrion organization”, “mitochondrial respiratory chain complex assembly” and “mitochondrion” in our hit list with adjusted *p*-values of 0.0009, 0.001, and 0.008, respectively. Other groups of hits corresponded to hypoxia signaling, autophagy, and lipid homeostasis. Taken together, this suggests that the presence of Tgl4 at this organelle interface is regulated by the metabolic state of the cell. Additionally, we identified eight genes from the membrane contact site database, representing a threefold enrichment to the expected amount given the fraction of the genome annotated, likely reflecting the tight interrelations within the cellular contact site network.

**Fig. 6:**
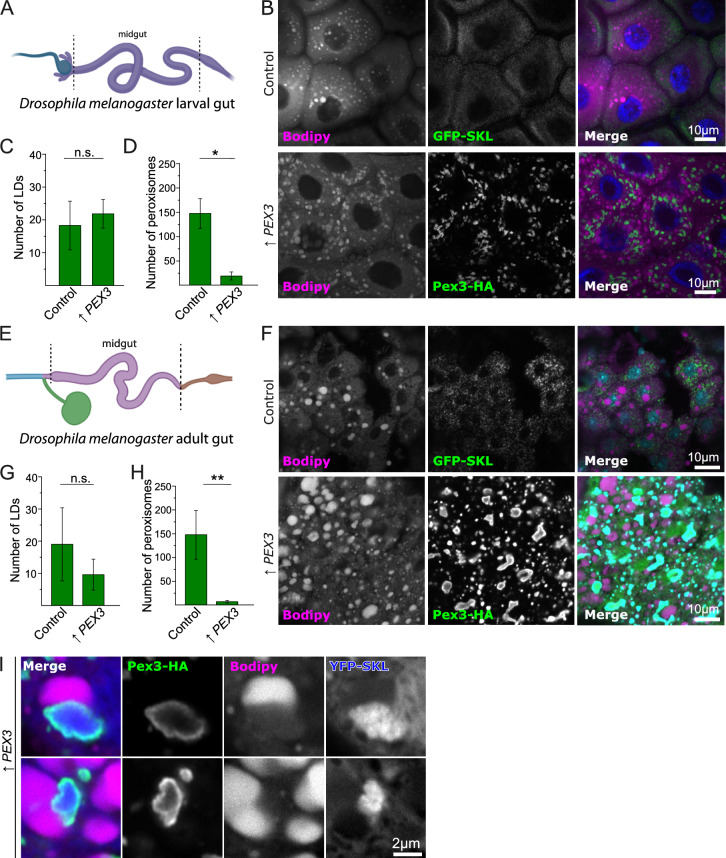
Overexpression of Pex3 leads to morphological changes in lipid droplets and peroxisomes in *Drosophila melanogaster*. (**A, E**) Diagrams of the cuts from *Drosophila melanogaster* that were analyzed. Larval midgut (A) and midgut from adult flies (B) were analyzed. (**B–D**) Representative images of control cells and strains overexpressing Pex3 in larval gut. Control cells express the GFP-SKL construct to visualize the lumen of the peroxisomes, while cells with overexpressed Pex3 also contain a GFP tag. Lipid droplets were stained with Bodipy and the nucleus with DAPI. Scale bars: 20 μm. Panels C and D show the quantification of the number of LDs (C) and peroxisomes (D). (**F–H**) Representative images of cells from the adult midgut from control flies and flies overexpressing Pex3. Control cells express the GFP-SKL construct to visualize the lumen of the peroxisomes, while cells with overexpressed Pex3 also contain a GFP tag. Lipid droplets were stained with Bodipy and the nucleus with DAPI. Scale bars: 20 μm. Panels G and H show the quantification of the number of LDs (C) and peroxisomes (D). (**I**) Representative Images of cells from adult flies midgut overexpressing Pex3-HA. Cells also express YFP-SKL to label the lumen of peroxisomes and lipid droplets were labeled with Bodipy. The enlarged organelles are observed in close proximity, with complementary morphological changes, suggesting the formation of membrane contact sites.

### The phenotype of overexpression of Pex3 is conserved in metazoans

Since Pex3 is a conserved protein, and many of its functions, like the incorporation of peroxisomal membrane proteins and the role in pexophagy are conserved in metazoans, we decided to test if the phenotype of induction of contact sites is also conserved. Thus, we overexpressed Pex3-HA in *Drosophila melanogaster* larvae in the midgut using the GAL/UAS system^58^, and we observed by fluorescence microscopy cells from the midgut, comparing them to cells expressing only GFP-SKL, to observe peroxisomes when Pex3 is not overexpressed (Fig. 6A and B). In the larval midgut, we observed that overexpression of Pex3 caused a reduction of the number of peroxisomes, as well as an increase in their size, whereas the number of lipid droplets was unaffected (Fig. 6B–D). This phenotype was exacerbated during development, with a drastic increase in peroxisomal and lipid droplet size in the adult midgut (Fig. 6E–H). We expressed Pex3-HA with another peroxisomal marker, YFP-SKL. Using Airyscan confocal microscopy, we found that Pex3-HA clearly labeled the surface of the enlarged peroxisome, while YFP was imported into the peroxisomal lumen by the peroxisomal targeting sequence, as expected (Fig. 6I). Furthermore, these enlarged peroxisomes were closely associated with lipid droplets as can be observed by the shape deformation of the organelles when they are next to each other. Thus, the phenotype closely resembles the one observed in yeast.

## Discussion

In this work, we have described that Pex3 overexpression induces the formation of peroxisome-peroxisome and peroxisome-lipid droplet contact sites. If we make the approximation that Pex3 expressed under the control of the *TEF1* promoter would have the levels of the Tef1 protein, and based on the integrated high throughput data in the protein abundance database PaxDB 5.0^59^, the overexpression system that we used would generate approximately a 200-fold increase in protein levels. We do not think of this as mimicking a physiological scenario, but rather as a tool for discovery, which has in the past proven useful to exacerbate one of the functions of a multifunctional protein over the others. For example, for the proteins Vps39 and Cvm1, overexpression allowed identification of their role as tethers of the vacuole-mitochondria contact site^60,61^. This role could later be confirmed at endogenous levels of the proteins either by addressing the deletion or separation-of-function mutants^61,62^. Our microscopy-based screens illustrate how useful this tool can be, since it already allowed the identification of Tgl4 as a player at peroxisomes-lipid droplet contact sites, and factors affecting its recruitment. In the future, our findings can establish the basis for screens designed to identify separation-of-function alleles, both for Pex3 and Tgl4, between their roles at these contact sites and their other biochemical functions. This type of mutants are invaluable tools when working with multi-functional proteins, and would be crucial for mechanistic studies into the function of the contact sites^63,64^.

The formation of the homotypic peroxisome contact sites is an interesting phenomenon that poses the question of the functionality of contacts among the same type of organelles. For many organelles, the existence of specialized subpopulations has been proposed. The formation of homotypic contacts could be related to this, and represent contacts enabling communication among different specialized subopulations of one organelle. In this case, they would be functionally analogous to heterotypic contact sites, facilitating communication between distinct compartments. Importantly, homotypic peroxisome contacts have been observed before in cultured mammalian cells^65,66^ and in tissues, even large-scale aggregates seem to be present^67^. Interestingly, homotypic contact sites have, to our knowledge, not been observed for most organelles. This could be related to the fact that, while most organelles can undergo homotypic fusion, it is thought that mature peroxisomes cannot fuse with each other^68^. Thus, homotypic contact sites could serve as a mechanism to re-organize the separation of functions among specialized subtypes, a process that in other organelles could be achieved by fusion.

Another interesting aspect is the ever-growing multi-functional nature of the Pex3 protein. This is another example of membrane contact site tethers being multi-functional proteins. It is a recurring observation that organelles use some proteins as flagposts, and that several different proteins which require interaction with that membrane use this same protein as a binding partner. The most canonical example is the VAP-family protein in the endoplasmic reticulum^36^, but this phenomenon is also apparent for other organelles, like Vac8 for the vacuole and Tom70 for mitochondria^39,42,43,69–71^. These last two examples, alike Pex3, are involved in different processes in addition to contact site tethering^72–74^. These multi-function proteins could aid the coordination or cross-regulation of different processes involving the same organelle. For example, the protein Vps39 is part of contact sites as well as a subunit of tethering complexes involved in vesicular transport, and the dynamics of the tethering complexes are linked to contact site formation^75^.

This phenomenon could also explain the difference between the overexpression phenotype observed in *H. polymorpha* and in *S. cerevisiae*. Pex3 might have acquired its multiple functions sequentially in evolution, apparently resulting in the formation of different Pex3-dependent contact sites in different species. We also observed that the peroxisomal structure formed upon Pex3 overexpression was often (55% of the cases) in proximity to the vacuole, but the phenotype was partial, prompting us to study the more penetrant phenotypes in peroxisome and lipid droplet clustering. The fraction of the peroxisomal-lipid droplet clusters found next to the vacuole was not affected by deletion of the known lipid droplet-vacuole tethers Ldo16/45, indicating that this proximity is generated either by a parallel tethering complex, or possibly rather depends on peroxisome tethering to the vacuole.

We also observed that Pex3 overexpression induced lipid droplet-peroxisomal contacts. These contacts have been observed in wild-type cells during growth in oleate^46,76^, and occasionally included protrusions of the peroxisomes into the lipid droplets, that were positive for the β-oxidation enzyme Pox1. The proposed role for this contact site is an optimization of lipolysis-derived transfer of fatty acids for β-oxidation. Our finding of the lipase Tgl4 as a possible tether is very much in line with this.

We observe that upon deletion of *TGL4*, the abundance of Pex3 overexpression-dependent peroxisome-lipid droplet contact sites decreases approximately by half. This suggests that tethering between these organelles does not exclusively depend on Tgl4, and the responsible tethers are yet to be described. Another possibility is that this residual clustering is related to the connection of both proteins with the endoplasmic reticulum as their place of biogenesis, even though we observed no effect of the deletion of Pex30, a protein that organizes the specific biogenesis subdomains^46^. Recently, in mammalian cells the hereditary spastic paraplegia protein M1 Spastin was shown to form a tethering complex between lipid droplets and peroxisomes through its interaction with the transporter ABCD1. This contact is thought to aid in the trafficking of fatty acids through a mechanism that involves membrane deformation by ESCRT-III^44^. The Tgl4-dependent contact site that we observe could have a different mechanism, and aid in fatty acid trafficking by local production of free fatty acids, as we observe several lipases to be enriched in the interface. In the future, it will be interesting to see how these different mechanisms interplay with each other, and to clarify if they operate in parallel in the same contact site, in structurally distinct ones, or in different species.

## Materials and methods

### Strains and plasmids

*Saccharomyces cerevisiae* strains were based on either BY4741 or W303. Genetic manipulations were carried out by homologous recombination of PCR-amplified cassettes as described in^77^. The Tgl4(S315G) mutant was generated by using CRISPR-Cas9 as described in^78^. For this, 500 ng of the plasmid pAGM218 and the oligonucleotide oAGM875 were transformed in the BY4741 strain. Plasmid pAGM218 was generated by PCR amplification of plasmid pCU5003 using primers oAGM873-874, and was confirmed by sequencing. We confirmed that the strain (AGMY1888) contained the appropriate mutation and no additional ones, by extracting genomic DNA, amplifying the region encoding for Tgl4 with primers oAGM803-804 and sequencing. All yeast strains used in this study are listed in Table S1.

The plasmid pAGM197 (pRS403 TEF1pr-BFP-SKL) was generated by Gibson Assembly, using primers oAGM839-840 and oAGM841-842 to amplify the BFP from plasmid CU5258 followed by an SKL tag, and the pRS403 backbone respectively. Purified PCR fragments were incubated with the Gibson Assembly master mix (New England Biolabs) for one hour at 55 °C. The resulting plasmid was transformed in competent *E. Coli* DH5α and clones were checked by sequencing.

The cytosolic domain of Pex3 was amplified from genomic DNA from a BY4741 strain, using primers oAGM470-471. The purified DNA fragment was digested with NotI and SacII enzymes and ligated in a pRS416 vector containing a TEF1 promoter and a N-terminal GFP tag, which was digested the same way. After this plasmid was confirmed by sequencing, it was digested with XhoI and SacI to ligate the fragment corresponding to TEF1pr-GFP-Pex3(CD) into a pRS403 backbone, to generate plasmid pAGM134 (pRS403 TEF1-GFP-Pex3(CD)).

The plasmid pAGM212 (pRS403 TEF1pr-AlfaTag-mKate2-Pex3(CD)) was done by Gibson Assembly. Primers oAGM748-749 were designed to amplify the cassette TEF1pr-GFP-Pex3(CD) from plasmid pAGM134 and adding an Alfa tag at the N-terminus. The purified DNA was incubated with a fragment containing the mKate2 sequence (amplified from pAGM042 with primers oAGM750-751) and the Gibson Assembly master mix (New England Biolabs) for one hour at 55 °C. The resulting plasmid was transformed in competent *E. Coli* DH5α and clones were checked by sequencing.

The plasmid pAGM210 (pRS403 TEF1pr-Pex3-mKate2) was also generated by Gibson Assembly. To obtain the corresponding fragments, primers oAGM851-852 were used to amplify the backbone (pRS403 TEF1pr) from plasmid pAGM134. Pex3 sequence was amplified from genomic DNA from a BY4741 strain with primers oAGM853-854. And last, mKate2 sequence was amplified by PCR using primers oAL855-856. All fragments were incubated with the Gibson Assembly master mix (New England Biolabs) for one hour at 55 °C. The resulting plasmid was transformed in competent *E. Coli* DH5α and clones were confirmed by sequencing.

The plasmid pAGM211 (pRS403 TEF1pr-Pex3 (W128K, L131K)-mKate2) was generated by PCR amplification of the entire plasmid pAGM210, using primers oAL831-832 which contain the desired punctual mutations. The presence of these mutations was confirmed by sequencing.

C-terminal Alfa tag was added to pAGM210 by PCR amplification of the plasmid. For this, primers oAGM910-911 were used, which include the sequence of the ALFA tag, to generate plasmid pAGM225 (pRS403 TEF1pr-Pex3-mKate2-Alfatag). pAGM226 (pRS403 TEF1pr-Pex3 (W128K, L131K)-mKate2-Alfatag) was generated in the same way but using plasmid pAGM211 as a source instead. Both plasmids were confirmed by sequencing. All pRS403-based plasmids were integrated into the HIS3 locus.

Plasmid pRS315-mCherry-SKL was a gift from Judith Müller (Institute of Molecular Genetics and Cell Biology, Ulm University) and PRS415-GFP-SKL from^79^. To obtain plasmid pAGM224, plasmid pRS315 GFP-SKL and pAGM197 were digested with SacI and XhoI. The purified fragment corresponding to TEF1pr-BFP-SKL was ligated into the pRS315 backbone. To stably integrate these constructs into the LEU2 locus, yeast transformations were done with DNA fragments amplified by PCR using primers oAGM090-091.

Plasmid pRS415 TGL4pr-Tgl4-msGFP2 was digested with ApaI and NotI restriction enzymes, taking the TGL4pr-Tgl4-msGFP2 construct. The purified DNA fragment was ligated to a pRS403 vector digested the same way. Plasmid pAGM242 (pRS403 TGL4pr-Tgl4-msGFP2) was confirmed by sequencing with oligonucleotide oAGM930.

All plasmids and oligonucleotides used in this study are listed in Tables S2 and S3, respectively.

### Fluorescence microscopy, image quantifications and statistical analysis

Cells were grown to logarithmic phase in yeast extract peptone medium containing glucose (YPD), or synthetic medium supplemented with essential amino acids (SDC). For experiments with enlarged Lipid Droplets and peroxisomes, cells were grown in SC-Oleate media as described in Yifrach et al., 2022, and washed with SC media before imaging. The lumen of the vacuole was stained by adding 20 µM 7-amino-4-chloromethylcoumarin (CMAC) dye (Invitrogen) to 0.5 OD Units and incubating them for 15 min at 30 °C with shaking, followed by one washing step in SDC medium. Proteins tagged with the HaloTag were labelled with the Janelia Fluor JFX650 ligand (Lavis Lab;^80^). Yeast cells (0.5 OD Units) were incubated with 1.6 µM of JFX650 for 15 min, followed by eight washing steps with SDC medium^81^. Lipid droplets were stained with Bodipy dye (Echelon Biosciences, through MoBiTec). Cells were incubated with 1 µg/ml of the dye for 15 min at 30 °C with shaking, followed by one wash with SDC medium.

In the majority of experiments, cells were imaged live in SDC medium on an Olympus IX-71 inverted microscope equipped with 100 × NA = 1.49 and 60 × NA = 1.40 objectives, an sCMOS camera (PCO, Kelheim, Germany), an InsightSSI illumination system, 4′,6-diamidino-2-phenylindole (DAPI), GFP, mCherry and Cy5 filters, and SoftWoRx software (Applied Precision, Issaquah, WA, USA). We used z-stacks with 200, 250 or 350 nm spacing for constrained-iterative deconvolution with the SoftWoRx software. For microscopy experiments of Tgl4 S315G mutant (Fig. 5E), cells were imaged on a Olympus microscope IX-71 equipped with 100 × oil NA = 1.45, 60 × oil NA = 1.42 and 60 × water NA = 1.2 objectives, a back-illuminated sCMOS camera (Hamamatsu ORCA Fusion-BT), a LED module (pe-800, CoolLED) and five different polychroic filter sets. The microscope is operated by Micro-Manager software version 2.0.3 nightly build (www.micro-manager.org). Images were deconvolved with Huygens Professional version 3.7.1 (Scientific Volume Imaging, The Netherlands, http://svi.nl).

All further image processing and quantification were performed using ImageJ (National Institutes of Health, Bethesda, MD, USA). One plane of the z-stack is shown in the figures.

The measurement of the size of lipid droplets was performed by making a maximum intensity Z-projection of all slices of the green channel (Bodipy staining), and binarizing the image with aYen threshold using the functionality in ImageJ. Regions of interest were manually established around each lipid droplet from 10 different cells in each triplicate. Only round-shaped lipid droplets were considered, to avoid assessing clusters of lipid droplets instead of single organelles. The number of pixels forming part of a lipid droplet was counted and converted to square micrometers. The localization of proteins at interfaces of contact sites (Figs. 2A, B and 5F) was quantified by performing a line profile around the lipid droplets observed in contact with a peroxisome. The intensity of signal in all channels was measured and values were manually aligned to the peak of the blue channel, corresponding to the BFP-SKL signal. Values were normalized to their maximum peak. For plots of Fig. 2A and B, values of the representative images are shown. For plots in Fig. 5G, averages and SD of ten cells (one peroxisome-lipid droplet interpase each) were plotted.

The colocalization measurement of the cytosolic domain of Pex3 with Lipid Droplets and peroxisomes (Fig. 3C) was performed by applying a maximum entropy threshold to all channels using Image J. Cells were manually defined as regions of interest (ROIs) and the Manders M1 and M2 coefficients for each cells were obtained with the JACoP plugin for ImageJ^82^.

For the distances measurement between the peroxisomes and mitochondria in Fig. 3F, the far red and green channels were analyzed. An Otsu threshold was applied to the raw data and 3D ROIs corresponding to peroxisomes and mitochondria were built with the 3D ROI manager plugin from the 3D ImageJ Suite and manually curated^83^. Border to border distances were measured using the 3D ROI manager between the different objects (peroxisomes and mitochondria) and the shortest distances for each peroxisome to mitochondria was plotted. The distributions of the distances between peroxisomes and mitochondria were compared using the Kolmogorov–Smirnov test, under the null hypothesis that the data from both samples present the same distribution, with the Origin 9 software. This test determined that the data distribution of both samples is significantly different (***, *P* < 0.0001). The images shown in the figure aremaximum intensity Z-projections of entire Z-stacks. Supplemental Fig. 2 shows different examples of each strain with a maximum intensity projection of all slices acquired.

### TurboID assay and mass spectrometry

To assess the molecular environment of overexpressed Pex3 by TurboID, three cultures of each strain, AGMY192 *(TEF1*pr-Pex3, labelled as “Control”) and AGMY575 (*TEF1pr*-Pex3-TurboID-V5, labelled as “TurboID”), were grown up to logarithmic phase in YPAD media. 100 µM of Biotin (Novabiochem) was added to the cultures and incubated for 3 h. 350 OD units were harvested, washed with sterile water and split into two vials. Cells were resuspended in 650 µl lysis buffer (20 mM TrisHCl pH 8, 1% sodium dedecilsulfate (SDS), 1 mM dithiotreitol (DTT)) and lysed twice in a Fastprep machine (MP Biomedicals) at 40 m/s for 40 s. Samples were treated with a heat shock (60 °C for 10 min) and after cooling down, 650 µl of buffer B were added (20 mM TrisHCl pH 8, 0,5% SDS, 8 M Urea, 1 mM DTT). Lysates were centrifuged at 20.000 g for 20 min. Supernatants belonging to the same sample were combined and protein concentration was measured using Bradford assay (Bio-Rad). 100 µl of slurry Streptavidin agarose beads (Thermo Fisher Scientific) were equilibrated for each sample which were added in equal protein concentrations. The samples were incubated for one hour at room temperature with rotation. The beads were centrifuged at 300×g for 1 min and washed four times with 1 ml wash buffer (20 mM TrisHCl pH 8, 0,75% SDS, 4 M Urea, 1 mM DTT) and four times with the same buffer without detergent. The samples were processed for mass spectrometry with the iST 96 × Kit (Preomics) according to manufacturer instructions.

For mass spectrometry analysis, reversed-phase chromatography was performed on a Thermo Ultimate 3000 RSLCnano system connected to a Q-ExactivePlus mass spectrometer (Thermo Fisher Scientific) through a nano-electrospray ion source. For peptide separation, 50 cm PepMap C18 easy spray columns (Thermo Fisher Scientific) with an inner diameter of 75 µm were used and kept at a temperature of 40 °C. The peptides were eluted from the column with a linear gradient of acetonitrile from 10 to 35% in 0.1% formic acid for 118 min at a constant flow rate of 300 nl/min, followed by direct electrospraying into the mass spectrometer. The mass spectra were acquired on the Q-Exactive Plus in a data-dependent mode to automatically switch between full scan MS and up to ten data-dependent MS/MS scans. The maximum injection time for full scans was 50 ms, with a target value of 3,000,000 at a resolution of 70,000 at m/z = 200. The ten most intense multiply charged ions (z = 2) from the survey scan were selected with an isolation width of 1.6 Th and fragments with higher energy collision dissociation with normalized collision energies of 27^84^. Target values for MS/MS were set at 100,000 with a maximum injection time of 80 ms at a resolution of 17,500 at m/z = 200. To avoid repetitive sequencing, the dynamic exclusion of sequenced peptides was set at 30 s.

The resulting MS and MS/MS spectra were analyzed using MaxQuant (version 1.6.0.13, https://www.maxquant.org/) utilizing the integrated ANDROMEDA search algorithms^85,86^. The peak lists were compared against local databases for *S. cerevisiae* (obtained from the *Saccharomyces* Genome database, Stanford University), with common contaminants added. The search included carbamidomethylation of cysteine as a fixed modification and methionine oxidation, N-terminal acetylation, and phosphorylation as variable modifications. The maximum allowed mass deviation was 6 ppm for MS peaks and 20 ppm for MS/MS peaks. The maximum number of missed cleavages was two. The false discovery rate was 0.01 on both the peptide and the protein level. The minimum required peptide length was six residues. Proteins with at least two peptides (one of them unique) were considered identified. The re-quant option of MaxQuant was disabled. The complete protein groups table is available as Table S6. The calculations and plots were performed using the Perseus software^87^.

### On-section CLEM tomography

#### High pressure freezing (HPF)

For high-pressure freezing, yeast cells were grown in YPD (1% yeast extract, 2% peptone, 2% glucose) medium to OD600 of 0.4–0.6. The suspension was concentrated by vacuum filtration onto a 0.45 µm membrane filter (Merck, HVWG04700), which was was placed onto an agar plate and the yeast paste was scraped using a pipette tip. Then, 3 µl of the concentrated paste were transferred into a 100 µm deep cavity of an aluminum planchette (Engineering Office M. Wohlwend GmbH, 241) until the cavity was overfilled. Subsequently, the flat side of a planchette (Engineering Office M. Wohlwend GmbH, 242) was placed on top and excess yeast paste was quickly removed. The finished assembly was immediately subjected to high-pressure freezing using a HPF Compact 03 (Engineering Office M. Wohlwend GmbH). Vitrified samples were stored in liquid nitrogen until further processing via freeze substitution.

#### Freeze substitution (FS)

Freeze substitution and Lowicryl embedding was performed as described in^88^ with slight modifications. Briefly, samples were freeze substituted in 0.1% uranyl acetate (Science Services, E22400) in anhydrous acetone (VWR, 83,683.230) for 24 h at -90 °C. Then, the temperature was raised to − 45 °C (5 °C/h) and the samples were washed three times with anhydrous acetone. Next, infiltration with increasing concentrations (10%, 25%, 50%, 75%) of Lowicryl HM20 in acetone (Science Services, PS14340) was carried out for 2 h each step. During the last two steps the temperature was raised by 10 °C each to − 35 °C and − 25 °C respectively. Afterwards 100% Lowicryl was exchanged three times in 10 h steps. Finally, polymerization was carried out via UV light for 24 h at − 25 °C and further 24 h at 20 °C. Polymerized sample blocks were taken out of the AFS and were ready for ultrathin sectioning.

#### On-section light microscopy

For light microscopy imaging, 200 mesh carbon film grids (Plano, S160) containing 250 nm thin sections of the samples were placed on a 20 µl drop of PBS, pH 8, on a 25 mm coverslip. The grids were sandwiched between another 25 mm coverslip and transferred to a custom-made holder. Z-stacks were acquired using an Olympus FV-3000 operated as a wide-field setup, equipped with an sCMOS camera (ORCA-Flash 4.0, Hamamatsu, Japan) and a 60 × oil immersion objective (PLAPON-SC NA 1.4). Overview images were taken with a 10 × objective (UPL SAPO NA 0.4) to facilitate later correlation within the transmission electron microscopy (TEM).

#### TEM tomography acquisition, correlation and segmentation

For TEM tomography, sections were labelled on both sides with 10 nm protein A gold fiducials. Sections were then contrasted with 3% uranyl acetate for 30 min and 2% lead citrate for 20 min in the LEICA EM AC20 and subsequently analysed with a TEM at 200 kV (JEM2100Plus, JEOL, Japan) equipped with a 20-megapixel EMSIS Xarosa CMOS camera (EMSIS, Germany). Regions of interest from the LM were relocated within the TEM and tilt series from + -65° with 1° increments were acquired using TEMography software (TEMography.com, JEOL, Japan). The tomograms were then reconstructed using the back projection algorithm in IMOD^89^. The tomography data were manually overlaid with the fluorescence signal from the LM using Adobe Photoshop.

### Pulldowns and Western blot

Cells were grown to logarithmic phase in yeast extract peptone medium containing glucose (YPD) and 100 OD600 units were harveste by centrifugation. The pellets were resuspended in lysis buffer (50 mM HEPES pH 7,4, 150 mM NaCl, 10% glycerol, 1% CHAPS, 1 mM PMSF, protease inhibitor cocktail FY (Serva) 1/100) and lysed twice in a FastPrep device (6 m/s for 40 s; MP Biomedicals), with a 5-min incubation on ice in between. The lysate was centrifuged at 20.000 xg for 20 min and 4 °C. 12.5 µl slurry GFP-Trap agarose beads (Chromotek) or Alfa agarose beads (ALFA selector ST, NanoTag Biotechnologies) were equilibrated with lysis buffer and the same protein concentration was added to the beads. Samples were incubated at 4 °C for 15 min with rotation. The beads were centrifuged at 300 × g for 1 min and washed twice with 1 ml lysis buffer and four times with 1 ml buffer without detergent. Proteins were eluted from the beads by incubating them with 1X Laemmli buffer (4% SDS, 0.05% bromophenol blue, 0.0625 M Tris, pH 7.4, 2.5% β-mercaptoethanol, and 10% glycerol) at 95 °C for 10 min.

Proteins were separated using SDS-PAGE in 10% Bis–Tris acrylamide/ bisacrylamide gels and transferred to a nitrocellulose membrane (GE Healthcare). The membranes were blocked for 30 min with PBS 5% milk and incubated with the first antibody at 4 °C overnight with gentle shaking. The membranes were washed three times with PBS, and once with TBS-Tween (0.5% (v/v) Tween 20) for 5 min. A fluorescent-dye-coupled secondary antibody (Thermo Fisher Scientific) was diluted 1:20,000 in PBS 5% milk and incubated at room temperature for 1 h. A Bio-Rad ChemiDoc MP imaging system was used to detect the fluorescent signal. The whole Western is shown in Supplemental Fig. 3D. Supplemental Fig. 3E shows the right side of the membrane (previously decorated with antibodies against the Alfa tag) after stripping and decorating with antibodies against GFP. For stripping, the membrane was washed with distilled water, incubated for 5 min with 1 M sodium hydroxide and washed again. Once the lack of fluorescent signal was confirmed by scanning, the membrane was blocked and incubated with the anti-GFP antibody as specified previously. The antibodies used are listed in Table S4.

### Detection of pexophagy via whole cell lysate and Western blot

All cells were grown overnight in SDC media as precultures. The logarithmic phase glucose (L.P.G.) samples, were diluted in SDC media and grown for 20hs without exceeding an OD_600_ = 0.3 and harvested. The pexophagy-inducing conditions samples (P.I.C) were diluted in synthetic media containing oleate as the carbon source (0.2% Oleate, 0.1% Tween-80) and grown for 20hs, then shifted to nitrogen starvation medium (without nitrogen source or amino acids) for 22hs and harvested. Whole cell lysates were generated by mechanical disruption using glass beads in lysis buffer (3 M urea, 1.875 mM EDTA-KOH p.H. 8.0, 1.2% (w/v) SDS, 37.5 mM Tris–HCl p.H. 6.8, 1.5% (v/v) glycerol. Bromophenol blue was added to the samples to a final concentration of 0.005% and 2-mercaptoethanol toafinal concentration of 0.5%. Equivalent amounts of each sample were subjected to SDS-PAGE, transferred to a nitrocellulose membrane and processed for Western blot as described in the previous section. The secondary antibody was coupled to horse-radish peroxidase, and the signal was detected using Sigma Lumi-Light Plus Western Blotting Substrate in an Azure 600 imaging System. The whole uncropped western blot membranes is shown in Supplemental Fig. 3A, and additional repetitions in Supplemental Fig. 3B and C. In the two other repetitions, the pre-cultures were included in the experiment (grown in glucose for the L.P.G condition and in oleate for the P.I.C. condition).

### Fly husbandry

Flies were reared on standard cornmeal food (130 g yarn agar, 248 g Baker’s yeast, 1223 g Cornmeal and 1.5 l sugar beet syrup in 20 l distilled water) and kept in a 25 °C incubator with light–dark-cycle. Fly lines used in this study were mex-Gal4 (kindly provided by the lab of Irene Miguel-Aliaga), UAS-Pex3-GFP, UAS-Pex3-HA (kindly provided by the lab of Reinhard Bauer) and UAS-GFP-SKL (Bloomington Drosophila stock center #28882). For studies in the larval gut, the following genotypes were used: w; mex-Gal4; UAS-GFP-SKL and w; mex-Gal4; UAS-GFP-SKL/UAS-Pex3-HA. For studies in the adult gut, the following genotypes were used: w; mex-Gal4; UAS-GFP-SKL, w; mex-Gal4; UAS-GFP-SKL/UAS-Pex3-HA and w; mex-Gal4; UAS-YFP-PTS1/UAS-Pex3-HA. Animals were reared on standard diet and analyzed as 3rd instar larvae or 5 day old adults (male and female), respectively.

### Imaging of Drosophila guts

Antibodies used in this study were α-GFP (Santa Cruz Biotechnology) and α-HA (Invitrogen), α-TOMM20 (Sigma-Aldrich). For immunohistochemistry, guts from 3rd instar larvae or adult flies were dissected in PBS and fixed for 1 h in 0.5% PBS-Tween20 and 4% formaldehyde. Tissue was washed with 0.5% PBS-Tween20 and blocked with donkey serum before incubation with the primary antibody (overnight at 4 °C). The tissue was washed in 0.1% PBS-Tween20 before incubation with BODIPY 581/591 (Thermo Scientific) and the secondary antibody at room temperature in the dark for 1 h. Secondary antibodies coupled to Alexa or Cyanine dyes were from Molecular probes. The tissue was washed and incubated for 5 min with DAPI (4',6-diamidino-2-phenylindole). For imaging, we used a Zeiss LSM 710 with a 25 × water lens (Plan-Neofluar, Zeiss), 40 × water lens (C-Apochromat, Zeiss), and 63 × water lens (Plan-Apochromat, Zeiss) and a Zeiss LSM 880 with Airyscan detector. We used ImageJ to quantify peroxisome and lipid droplet number and area from at least 3 individual cells from different experiments.

### Library generation and high-throughput microscopy

All yeast manipulations were performed in high-density format (384–1,536 strains per plate) using a RoToR bench-top colony array instrument (Singer Instruments). In order to find key proteins involved in the formation of the peroxisome-peroxisome or peroxisome-lipid droplet contact sites (Fig. 5A), strain AGMY1303, with an overexpression of Pex3 (*TEF1*pr-Pex3) and a peroxisomal marker (Pex14-2xmKate2) was crossed with a genome-wide library of deletion^47^ and hypomorphic allele^48^ strains, by the synthetic genetic array method^49,50^. For analysis of Tgl4 localization (Supplemental Fig. 6), strain yMB1326 (*TEF2*pr-Pex3 Erg6-mCh BFP-SKL Tgl4-GFP) was crossed with the same mutant collections.

Cells were mated on rich medium plates, diploids were selected and sporulation was induced by incubating the cells for five to eight days on nitrogen starvation media plates. Haploid cells were selected on 50 mg/L Canavanine and 50 mg/L Thialysine. Finally, haploid cells containing the combination of all desired manipulations were selected. A subset of strains was verified by microscopy and confirmed by PCR.

For the automated imaging of the obtained libraries, cells were first transferred from agar plates into 384 well plates for growth in liquid medium. For the screen for genes involved in formation of lipid droplet-peroxisome contacts (Fig. 5A), cultures were grown overnight at 30 °C in SDC. A JANUS liquid handler (PerkinElmer) connected to the incubator was used to dilute the strains to an OD600 of ∼0.2, and plates were incubated at 30 °C for 4 h. Cells were washed and fresh SDC media was added containing 20 µM 7-amino-4-chloromethylcoumarin (CMAC) dye and 1 µg/ml of Bodipy dye, followed by half an hour incubation and another wash step. For the Tgl4-GFP screen (Supplemental Fig. 6), liquid handling was performed using a MicroPro 300 liquid handler. Following the same overnight culture as the previous screen, cells were grown in the presence of 0.2% oleate in synthetic medium for 24 h for lipid droplet enlargement.

Strains were then transferred by a liquid handler into glass-bottom 384-well microscope plates (Matrical Bioscience) coated with concanavalin A (Sigma-Aldrich) and incubated for 20 min to allow adhesion of cells to the bottom of the plates. Afterwards, wells were washed twice with SDC medium (SC medium for oleate treated cells) to remove non-adherent cells leaving a cell monolayer. Plates were then transferred to an Olympus automated inverted fluorescence microscope system. In the screen for lipid droplet-peroxisome contact site proteins (Fig. 5A), cells were imaged in SDC at 18–20 °C using a 60 × air lens (NA 0.9) and with an ORCA-ER charge-coupled device camera (Hamamatsu), using ScanR software. In the Tgl4-GFP screen (Supplemental Fig. 6), cells were imaged in SC medium at 18–20 °C using a 60 × air lens (NA 0.9) and with an ORCA-flash4.0 camera (Hamamatsu), using ScanR software. After acquisition, images were manually reviewed using ImageJ (National Institutes of Health).

## Supplementary Information

Supplementary Information 1.

Supplementary Information 2.

Supplementary Information 3.

Supplementary Information 4.

Supplementary Information 5.

Supplementary Information 6.

Supplementary Video 1.

Supplementary Video 2.

Supplementary Information 7.

Supplementary Information 8.

## Data Availability

The proteomics dataset generated in this study is available via ProteomeXchange with identifier PXD063514. Other datasets generated during the current study are available from the corresponding author on reasonable request.

## References

[1] Wanders, R. J. A., Baes, M., Ribeiro, D., Ferdinandusse, S. & Waterham, H. R. The physiological functions of human peroxisomes. *Physiol. Rev.* **103**, 957–1024 (2023). 35951481 10.1152/physrev.00051.2021

[2] Scorrano, L. et al. Coming together to define membrane contact sites. *Nat. Commun.* **10**, 1–11 (2019). 30894536 10.1038/s41467-019-09253-3 PMC6427007

[3] Eisenberg-Bord, M., Shai, N., Schuldiner, M. & Bohnert, M. A tether is a tether is a tether: Tethering at membrane contact sites. *Dev. Cell* **39**, 395–409 (2016). 27875684 10.1016/j.devcel.2016.10.022

[4] Prinz, W. A., Toulmay, A. & Balla, T. The functional universe of membrane contact sites. *Nat. Rev. Mol. Cell Biol.* **21**, 7–24 (2020). 31732717 10.1038/s41580-019-0180-9 PMC10619483

[5] Valm, A. M. et al. Applying systems-level spectral imaging and analysis to reveal the organelle interactome. *Nature* **546**, 162–167 (2017). 28538724 10.1038/nature22369 PMC5536967

[6] Shai, N. *et al.* Systematic mapping of contact sites reveals tethers and a function for the peroxisome-mitochondria contact. *Nat. Commun.* **9** (2018). 10.1038/s41467-018-03957-8 PMC5932058 29720625

[7] Kakimoto, Y. *et al.* Visualizing multiple inter-organelle contact sites using the organelle-Targeted split-GFP system. *Sci. Rep.* **8** (2018). 10.1038/s41598-018-24466-0 PMC5906596 29670150

[8] Voeltz, G. K., Sawyer, E. M., Hajnóczky, G. & Prinz, W. A. Making the connection: How membrane contact sites have changed our view of organelle biology. *Cell* **187**, 257–270 (2024). 38242082 10.1016/j.cell.2023.11.040 PMC11830234

[9] Shai, N., Schuldiner, M. & Zalckvar, E. No peroxisome is an island—Peroxisome contact sites. *Biochim. Biophys. Acta Mol. Cell Res.* **1863**, 1061–1069 (2016). 10.1016/j.bbamcr.2015.09.016 PMC4869879 26384874

[10] David, C. et al. A combined approach of quantitative interaction proteomics and live-cell imaging reveals a regulatory role for endoplasmic reticulum (ER) Reticulon Homology Proteins in peroxisome biogenesis. *Mol. Cell. Proteomics* **12**, 2408–2425 (2013). 23689284 10.1074/mcp.M112.017830 PMC3769320

[11] Yan, M., Rachubinski, D. A., Joshi, S., Rachubinski, R. A. & Subramani, S. Dysferlin domain-containing proteins, Pex30p and Pex31p, localized to two compartments, control the number and size of oleate-induced peroxisomes in pichia pastoris. *Mol. Biol. Cell* **19**, 885–898 (2008). 18094040 10.1091/mbc.E07-10-1042 PMC2262989

[12] Knoblach, B. et al. An ER-peroxisome tether exerts peroxisome population control in yeast. *EMBO J.* **32**, 2439–2453 (2013). 23900285 10.1038/emboj.2013.170 PMC3770948

[13] Burnett, S. F., Farré, J. C., Nazarko, T. Y. & Subramani, S. Peroxisomal Pex3 activates selective autophagy of peroxisomes via interaction with the pexophagy receptor Atg30. *J. Biol. Chem.* **290**, 8623–8631 (2015). 25694426 10.1074/jbc.M114.619338 PMC4375511

[14] Fang, Y., Morrell, J. C., Jones, J. M. & Gould, S. J. PEX3 functions as a PEX19 docking factor in the import of class I peroxisomal membrane proteins. *J. Cell Biol.* **164**, 863–875 (2004). 15007061 10.1083/jcb.200311131 PMC2172291

[15] Motley, A. M., Nuttall, J. M. & Hettema, E. H. Pex3-anchored Atg36 tags peroxisomes for degradation in *Saccharomyces cerevisiae*. *EMBO J.* **31**, 2852–2868 (2012). 22643220 10.1038/emboj.2012.151 PMC3395097

[16] Sato, Y. et al. Characterization of the interaction between recombinant human peroxin Pex3p and Pex19p: Identification of TRP-104 in Pex3p as a critical residue for the interaction. *J. Biol. Chem.* **283**, 6136–6144 (2008). 18174172 10.1074/jbc.M706139200

[17] Sato, Y. et al. Structural basis for docking of peroxisomal membrane protein carrier Pex19p onto its receptor Pex3p. *EMBO J.* **29**, 4083–4093 (2010). 21102411 10.1038/emboj.2010.293 PMC3018794

[18] Schmidt, F. et al. The role of conserved PEX3 regions in PEX19-binding and peroxisome biogenesis. *Traffic* **13**, 1244–1260 (2012). 22624858 10.1111/j.1600-0854.2012.01380.x

[19] Yamashita, S. I., Abe, K., Tatemichi, Y. & Fujiki, Y. The membrane peroxin PEX3 induces peroxisome-ubiquitination-linked pexophagy. *Autophagy* **10**, 1549–1564 (2014). 25007327 10.4161/auto.29329 PMC4206534

[20] Hulmes, G. E. *et al.* The Pex3-Inp1 complex tethers yeast peroxisomes to the plasma membrane. *J. Cell Biol.* **219** (2020). 10.1083/jcb.201906021 PMC7659723 32970792

[21] Wu, H. et al. Peroxisome development in yeast is associated with the formation of Pex3-dependent peroxisome-vacuole contact sites. *Biochim. Biophys. Acta Mol. Cell Res.* **1866**, 349–359 (2019). 30595161 10.1016/j.bbamcr.2018.08.021

[22] Fernández-Suárez, M., Scott Chen, T. & Ting, A. Y. Protein-protein interaction detection in vitro and in cells by proximity biotinylation. *NIH Public Access* **23**, 1–7 (2008). 10.1021/ja801445p PMC2635094 18582056

[23] Branon, T. C. et al. Efficient proximity labeling in living cells and organisms with TurboID. *Nat. Biotechnol.* **176**, 139–148 (2018). 10.1038/nbt.4201 PMC6126969 30125270

[24] Erdmann, R. & Blobel, G. Giant peroxisomes in oleic acid-induced *Saccharomyces cerevisiae* lacking the peroxisomal membrane protein Pmp27p. *J. Cell Biol.* **128**, 509–523 (1995). 7860627 10.1083/jcb.128.4.509 PMC2199900

[25] Yifrach, E. et al. Systematic multi-level analysis of an organelle proteome reveals new peroxisomal functions. *Mol. Syst. Biol.* **18**, 1–21 (2022). 10.15252/msb.202211186 PMC9513677 36164978

[26] Sandager, L. et al. Storage lipid synthesis is non-essential in yeast. *J. Biol. Chem.* **277**, 6478–6482 (2002). 11741946 10.1074/jbc.M109109200

[27] Höhfeld, J., Veenhuis, M. & Kunau, W. H. PAS3, a Saccharomyces cerevisiae gene encoding a peroxisomal integral membrane protein essential for peroxisome biogenesis. *J. Cell Biol.* **114**, 1167–1178 (1991). 1894692 10.1083/jcb.114.6.1167 PMC2289127

[28] Soukupovaa, M., Sprenger, C., Gorgas, K., Kunau, W.-H. & Dodtl, G. Identification and Characterization of the Human Peroxin PEX3. *Eur. J. Cell Biol.* **78**. http://www.urbanfischer.de/journals/ejcb (1999). 10.1016/S0171-9335(99)80078-8 10430017

[29] Schmidt, F. et al. Insights into peroxisome function from the structure of PEX3 in complex with a soluble fragment of PEX19. *J. Biol. Chem.* **285**, 25410–25417 (2010). 20554521 10.1074/jbc.M110.138503 PMC2919104

[30] Götzke, H. et al. The ALFA-tag is a highly versatile tool for nanobody-based bioscience applications. *Nat. Commun.* **10**, 1–12 (2019). 31562305 10.1038/s41467-019-12301-7 PMC6764986

[31] Torggler, R., Papinski, D. & Kraft, C. Assays to monitor autophagy in *Saccharomyces cerevisiae*. *Cells*10.3390/cells6030023 (2017). 28703742 10.3390/cells6030023 PMC5617969

[32] Kolitsida, P. *et al.* The pyruvate dehydrogenase complex regulates mitophagic trafficking and protein phosphorylation. *Life Sci. Alliance* **9** (2023). 10.26508/lsa.202302149 PMC10345312 37442609

[33] Yu, J. *et al.* Autophagy-related protein UvAtg7 contributes to mycelial growth, virulence, asexual reproduction and cell stress response in rice false smut fungus Ustilaginoidea virens. *Fungal Genetics Biol.* **159** (2022). 10.1016/j.fgb.2022.103668 35041987

[34] Hettema, E. H., Girzalsky, W., Van Den Berg, M., Erdmann, R. & Distel, B. Saccharomyces cerevisiae Pex3p and Pex19p are required for proper localization and stability of peroxisomal membrane proteins. *EMBO J.* **19**, 223–233 (2000). 10637226 10.1093/emboj/19.2.223 PMC305556

[35] Levine, T. P. & Munro, S. Dual targeting of Osh1p, a yeast homologue of oxysterol-binding protein, to both the Golgi and the nucleus-vacuole junction. *Mol. Biol. Cell* **12**, 1633–1644 (2001). 11408574 10.1091/mbc.12.6.1633 PMC37330

[36] Loewen, C. J. R., Roy, A. & Levine, T. P. A conserved ER targeting motif in three families of lipid binding proteins and in Opi1p binds VAP. *EMBO J.* **22**, 2025–2035 (2003). 12727870 10.1093/emboj/cdg201 PMC156073

[37] Kvam, E. & Goldfarb, D. S. Nvj1p is the outer-nuclear-membrane receptor for oxysterol-binding protein homolog Osh1p in *Saccharomyces cerevisiae*. *J. Cell Sci.* **117**, 4959–4968 (2004). 15367582 10.1242/jcs.01372

[38] Elbaz-Alon, Y. et al. Lam6 regulates the extent of contacts between organelles. *Cell Rep.* **12**, 7–14 (2015). 26119743 10.1016/j.celrep.2015.06.022 PMC4518459

[39] Murley, A. et al. Ltc1 is an ER-localized sterol transporter and a component of ER-mitochondria and ER-vacuole contacts. *J. Cell Biol.* **209**, 539–548 (2015). 25987606 10.1083/jcb.201502033 PMC4442815

[40] Toulmay, A. & Prinz, W. A. A conserved membrane-binding domain targets proteins to organelle contact sites. *J. Cell Sci.* **125**, 49–58 (2012). 22250200 10.1242/jcs.085118 PMC3269022

[41] Liu, L. K., Choudhary, V., Toulmay, A. & Prinz, W. A. An inducible ER-Golgi tether facilitates ceramide transport to alleviate lipotoxicity. *J. Cell Biol.* **216**, 131–147 (2017). 28011845 10.1083/jcb.201606059 PMC5223604

[42] Diep, D. T. V. et al. A metabolically controlled contact site between vacuoles and lipid droplets in yeast. *Dev. Cell* **59**, 740-758.e10 (2024). 38367622 10.1016/j.devcel.2024.01.016

[43] Álvarez-Guerra, I. et al. LDO proteins and Vac8 form a vacuole-lipid droplet contact site to enable starvation-induced lipophagy in yeast. *Dev. Cell* **59**, 759-775.e5 (2024). 38354739 10.1016/j.devcel.2024.01.014

[44] Chang, C. L. et al. Spastin tethers lipid droplets to peroxisomes and directs fatty acid trafficking through ESCRT-III. *J. Cell Biol.* **218**, 2583–2599 (2019). 31227594 10.1083/jcb.201902061 PMC6683741

[45] Joshi, A. S. et al. A family of membrane-shaping proteins at ER subdomains regulates pre-peroxisomal vesicle biogenesis. *J. Cell Biol.* **215**, 515–529 (2016). 27872254 10.1083/jcb.201602064 PMC5119935

[46] Joshi, A. S. et al. Lipid droplet and peroxisome biogenesis occur at the same ER subdomains. *Nat. Commun.* **9**, 1–12 (2018). 30054481 10.1038/s41467-018-05277-3 PMC6063926

[47] Giaever, G. *et al. Functional Profiling of the Saccharomyces Cerevisiae Genome*. http://www.kegg.com (2002). 10.1038/nature00935 12140549

[48] Breslow, D. K. *et al. A Comprehensive Strategy Enabling High-Resolution Functional Analysis of the Yeast Genome*. 10.1038/nmeth.1234 PMC2756093 18622397

[49] Cohen, Y. & Schuldiner, M. Advanced methods for high-throughput microscopy screening of genetically modified yeast libraries. In *Methods in Molecular Biology* Vol. 781 127–159 (Humana Press Inc., 2011). 10.1007/978-1-61779-276-2_8 21877281

[50] Hin, A., Tong, Y. & Boone, C. Synthetic genetic array analysis in *Saccharomyces Cerevisiae*. *From: Methods in Molecular Biology* vol. 313 (2006). 10.1385/1-59259-958-3:171 16118434

[51] Rajakumari, S., Rajasekharan, R. & Daum, G. Triacylglycerol lipolysis is linked to sphingolipid and phospholipid metabolism of the yeast *Saccharomyces cerevisiae*. *Biochim. Biophys. Acta Mol. Cell Biol. Lipids* **1801**, 1314–1322 (2010). 10.1016/j.bbalip.2010.08.004 20727985

[52] Athenstaedt, K. & Daum, G. Tgl4p and Tgl5p, two triacylglycerol lipases of the yeast Saccharomyces cerevisiae are localized to lipid particles. *J. Biol. Chem.* **280**, 37301–37309 (2005). 16135509 10.1074/jbc.M507261200

[53] Athenstaedt, K. & Daum, G. YMR313c/TGL3 encodes a novel triacylglycerol lipase located in lipid particles of Saccharomyces cerevisiae. *J. Biol. Chem.* **278**, 23317–23323 (2003). 12682047 10.1074/jbc.M302577200

[54] Jandrositz, A. et al. The lipid droplet enzyme Tgl1p hydrolyzes both steryl esters and triglycerides in the yeast, *Saccharomyces cerevisiae*. *Biochim. Biophys. Acta Mol. Cell Biol. Lipids* **1735**, 50–58 (2005). 10.1016/j.bbalip.2005.04.005 15922657

[55] Thoms, S., Debelyy, M. O., Connerth, M., Daum, G. & Erdmann, R. The putative *Saccharomyces cerevisiae* hydrolase Ldh1p is localized to lipid droplets. *Eukaryot. Cell* **10**, 770–775 (2011). 21478430 10.1128/EC.05038-11 PMC3127662

[56] Köffel, R., Tiwari, R., Falquet, L. & Schneiter, R. The *Saccharomyces cerevisiae* YLL012/YEH1, YLR020/YEH2, and TGL1 Genes Encode a Novel Family of Membrane-Anchored Lipases That Are Required for Steryl Ester Hydrolysis. *Mol. Cell Biol.* **25**, 1655–1668 (2005). 15713625 10.1128/MCB.25.5.1655-1668.2005 PMC549362

[57] Kurat, C. F. et al. Obese yeast: Triglyceride lipolysis is functionally conserved from mammals to yeast. *J. Biol. Chem.* **281**, 491–500 (2006). 16267052 10.1074/jbc.M508414200

[58] Brand, H. & Perrimon, N. *Targeted Gene Expression as a Means of Altering Cell Fates and Generating Phenotypes* (1993). 10.1242/dev.118.2.401 8223268

[59] Huang, Q., Szklarczyk, D., Wang, M., Simonovic, M. & Mering, C. von. PaxDb 5.0: Curated protein quantification data suggests adaptive proteome changes in yeasts. *Mol. Cell. Proteom.* **22** (2023). 10.1016/j.mcpro.2023.100640 PMC10551891 37659604

[60] Hönscher, C. et al. Cellular metabolism regulates contact sites between vacuoles and mitochondria. *Dev. Cell* **30**, 86–94 (2014). 25026035 10.1016/j.devcel.2014.06.006

[61] Bisinski, D. D. *et al.* Cvm1 is a component of multiple vacuolar contact sites required for sphingolipid homeostasis. *J. Cell Biol.* **221** (2022). 10.1083/jcb.202103048 PMC9247719 35766971

[62] González Montoro, A. et al. Vps39 interacts with Tom40 to establish one of two functionally distinct vacuole-mitochondria contact sites. *Dev. Cell* **45**, 621-636.e7 (2018). 29870720 10.1016/j.devcel.2018.05.011

[63] Gurevich, V. V. & Gurevich, E. V. Analyzing the roles of multi-functional proteins in cells: The case of arrestins and GRKs. *Crit. Rev. Biochem. Mol. Biol.* **50**, 440–452. 10.3109/10409238.2015.1067185 (2015). 26453028 10.3109/10409238.2015.1067185 PMC4852696

[64] Lubin, J. W., Rao, T., Mandel, E. K., Wuttke, D. S. & Lundblad, V. Dissecting protein function: An efficient protocol for identifying separation-of-function mutations that encode structurally stable proteins. *Genetics* **193**, 715–725 (2013). 23307900 10.1534/genetics.112.147801 PMC3583993

[65] Schrader, M., King, S. J., Stroh, T. A. & Schroer, T. A. Real time imaging reveals a peroxisomal reticulum in living cells. **3671**, 3663–3671 (2000). 10.1242/jcs.113.20.3663 11017881

[66] Bonekamp, N. A., Sampaio, P., de Abreu, F. V., Lüers, G. H. & Schrader, M. Transient complex interactions of mammalian peroxisomes without exchange of matrix or membrane marker proteins. *Traffic* **13**, 960–978 (2012). 22435684 10.1111/j.1600-0854.2012.01356.x

[67] Gorgas, K. & Zaar, K. Anatomy and embryology peroxisomes in sebaceous glands III. Morphological similarities of peroxisomes with smooth endoplasmic reticulum and Golgi stacks in the circumanal gland of the dog* **. *Anat. Embryol.* **169** (1984). 10.1007/BF00300582 6721224

[68] Mast, F. D., Rachubinski, R. A. & Aitchison, J. D. Peroxisome prognostications: Exploring the birth, life, and death of an organelle. *J. Cell Biol.* **219**, 1–13 (2020). 10.1083/jcb.201912100 PMC7054992 32211898

[69] Pan, X. et al. Nucleus-vacuole junctions in *Saccharomyces cerevisiae* are formed through the direct interaction of Vac8p with Nvj1p. *Mol. Biol. Cell* **11**, 2445–2457 (2000). 10888680 10.1091/mbc.11.7.2445 PMC14931

[70] Filadi, R. et al. TOM70 sustains cell bioenergetics by promoting IP3R3-mediated ER to mitochondria Ca^2+^ transfer. *Curr. Biol.* **28**, 369-382.e6 (2018). 29395920 10.1016/j.cub.2017.12.047

[71] Hollenstein, D. M. *et al.* Vac8 spatially confines autophagosome formation at the vacuole in S. Cerevisiae. *J. Cell Sci.* **132** (2019). 10.1242/jcs.235002 PMC6899017 31649143

[72] Veit, M., Laage, R., Dietrich, L., Wang, L. & Ungermann, C. Vac8p release from the SNARE complex and its palmitoylation are coupled and essential for vacuole fusion. *EMBO J.* **20**, 3145–3155 (2001). 11406591 10.1093/emboj/20.12.3145 PMC150195

[73] Wang, Y. X., Catlett, N. L. & Weisman, L. S. Vac8p, a vacuolar protein with armadillo repeats, functions in both vacuole inheritance and protein targeting from the cytoplasm to vacuole. *J. Cell Biol.* **140**, 1063–1074 (1998). 9490720 10.1083/jcb.140.5.1063 PMC2132703

[74] Schmidt, O., Pfanner, N. & Meisinger, C. Mitochondrial protein import: From proteomics to functional mechanisms. *Nat. Rev. Mol. Cell Biol.* **11**, 655–667. 10.1038/nrm2959 (2010). 20729931 10.1038/nrm2959

[75] González Montoro, A. et al. Subunit exchange among endolysosomal tethering complexes is linked to contact site formation at the vacuole. *Mol. Biol. Cell* **32**, br14 (2021). 34668759 10.1091/mbc.E21-05-0227 PMC8694092

[76] Binns, D. et al. An intimate collaboration between peroxisomes and lipid bodies. *J. Cell Biol.* **173**, 719–731 (2006). 16735577 10.1083/jcb.200511125 PMC2063889

[77] Janke, C. et al. A versatile toolbox for PCR-based tagging of yeast genes: New fluorescent proteins, more markers and promoter substitution cassettes. *Yeast* **21**, 947–962 (2004). 15334558 10.1002/yea.1142

[78] Generoso, W. C., Gottardi, M., Oreb, M. & Boles, E. Simplified CRISPR-Cas genome editing for *Saccharomyces cerevisiae*. *J. Microbiol. Methods* **127**, 203–205 (2016). 27327211 10.1016/j.mimet.2016.06.020

[79] Schäfer, A., Kerssen, D., Veenhuis, M., Kunau, W.-H. & Schliebs, W. Functional Similarity between the Peroxisomal PTS2 Receptor Binding Protein Pex18p and the N-Terminal Half of the PTS1 Receptor Pex5p. *Mol. Cell Biol.* **24**, 8895–8906 (2004). 15456864 10.1128/MCB.24.20.8895-8906.2004 PMC517879

[80] Grimm, J. B. et al. A general method to improve fluorophores using deuterated auxochromes. *JACS Au* **1**, 690–696 (2021). 34056637 10.1021/jacsau.1c00006 PMC8154212

[81] Day, K. J., Casler, J. C. & Glick, B. S. Budding yeast has a minimal endomembrane system. *Dev. Cell* **44**, 56-72.e4 (2018). 29316441 10.1016/j.devcel.2017.12.014 PMC5765772

[82] Bolte, S. & Cordelieres, F. P. A guided tour into subcellular colocalization analysis in light microscopy. *J. Microsc.* **224** (2006). 10.1111/j.1365-2818.2006.01706.x 17210054

[83] Ollion, J., Cochennec, J., Loll, F., Escudé, C. & Boudier, T. TANGO: A generic tool for high-throughput 3D image analysis for studying nuclear organization. *Bioinformatics* **29**, 1840–1841 (2013). 23681123 10.1093/bioinformatics/btt276 PMC3702251

[84] Olsen, J. V. et al. Higher-energy C-trap dissociation for peptide modification analysis. *Nat. Methods* **4**, 709–712 (2007). 17721543 10.1038/nmeth1060

[85] Cox, J. & Mann, M. MaxQuant enables high peptide identification rates, individualized p.p.b.-range mass accuracies and proteome-wide protein quantification. *Nat. Biotechnol.* **26**, 1367–1372 (2008). 19029910 10.1038/nbt.1511

[86] Cox, J. et al. Andromeda: A peptide search engine integrated into the MaxQuant environment. *J. Proteome Res.* **10**, 1794–1805 (2011). 21254760 10.1021/pr101065j

[87] Tyanova, S., Temu, T. & Cox, J. The MaxQuant computational platform for mass spectrometry-based shotgun proteomics. *Nat. Protoc.* **11**, 2301–2319 (2016). 27809316 10.1038/nprot.2016.136

[88] Ader, N. R. & Kukulski, W. triCLEM: Combining high-precision, room temperature CLEM with cryo-fluorescence microscopy to identify very rare events. *Methods Cell Biol.* **140**, 303–320 (2017). 28528638 10.1016/bs.mcb.2017.03.009

[89] Kremer, J. R., Mastronarde, D. N. & Mcintosh, J. R. *Computer Visualization of Three-Dimensional Image Data Using IMOD*. (1996). 10.1006/jsbi.1996.0013 8742726

