# Supplementary material for: Pex3 promotes formation of peroxisome-peroxisome and peroxisome-lipid droplet contact sites
Source: Sci Rep. 2025 Jul 8;15:24480. doi: 10.1038/s41598-025-07934-2 (PMC12238565; doi:10.1038/s41598-025-07934-2)
Supplement: Supplementary file 1 — Supplementary Information 1. [file 41598_2025_7934_MOESM1_ESM.pdf]

Amado et al 2025 - Supplementary Figure 1

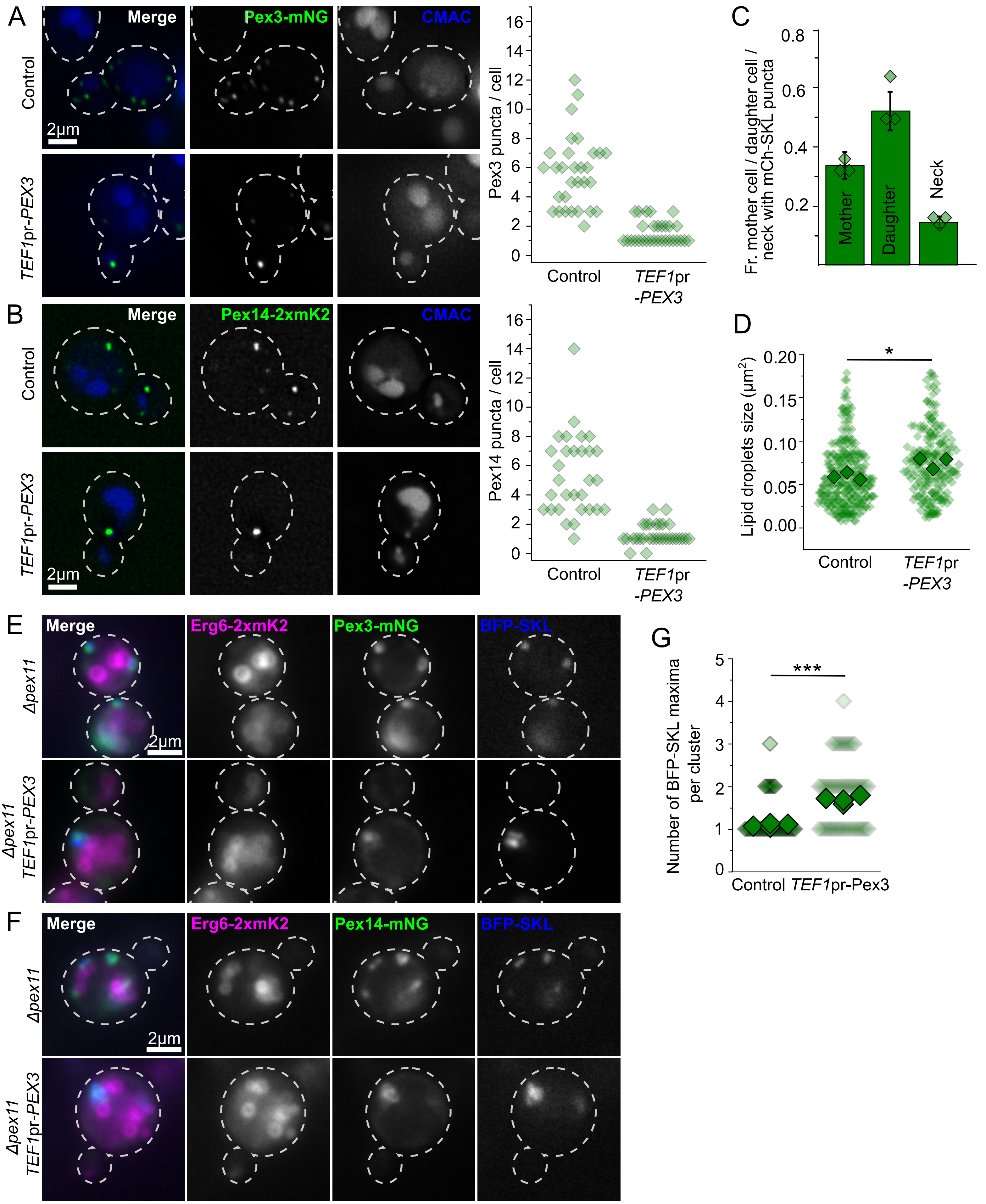

Supplemental Figure 1: Overexpression of Pex3 reduces the number of puncta of different peroxisomal markers.

A) Representative pictures of a strain expressing Pex3 fused to mNeonGreen to visualize the peroxisomes, either at endogenous levels (Control) or overexpressed (*TEF1*pr-*PEX3*) and the vacuolar lumen stained with CMAC. Cell outlines are shown as white dashed lines. Scale bar: 2 µm. The quantification of the amount of Pex3 puncta per cell is shown to the right. 30 cells from a single experiment were analyzed for each condition. Diamonds correspond to individual cells.

B) Representative pictures of a strain expressing Pex14 fused to 2xmKate2 to visualize the peroxisomes, either with Pex3 at endogenous levels (Control) or overexpressed (*TEF1*pr-*PEX3*) and the vacuolar lumen stained with CMAC. Cell outlines are shown as white dashed lines. Scale bar: 2 µm. The quantification of the amount of Pex14 puncta per cell is shown to the right. 30 cells from a single experiment were analyzed for each condition. Diamonds correspond to individual cells.

C) Fraction of mother cells, daughter cells or cell neck regions with mCherry-SKL puncta. The bars represent an average and standard deviation of three independent experiments, shown as diamonds.

D) Quantification of the size of lipid droplets, either with endogenous levels of Pex3 (Control) or upon Pex3 overexpression (*TEF1*pr-*PEX3*). Three independent experiments were performed and lipid droplets sizes from 10 cells were analyzed for each experiment and condition. Each small diamond represents a single cell, and the bigger ones represent the average of each of three independent experiments. The different strains were compared using an unpaired two-tailed Student's t-test. * $P < 0.05$.

E-F) Enlarged peroxisomes and LDs reveal that the structures contain several maxima for peroxisome lumen signal, with peroxisomal membrane between them. Representative pictures of control strains or strains with overexpressed Pex3 (*TEF1*pr-*PEX3*), expressing the BFP-SKL construct to visualize the lumen of the peroxisomes, Erg6-2xmKate2 marking the lipid droplet monolayer, and either Pex3 (E) or Pex14 (F) tagged with mNeonGreen as markers of the peroxisomal membrane. To produce enlarged peroxisomes and lipid droplets, the cells contain a deletion of *PEX11*, and were grown with oleate as the sole carbon source for 20hs. Cell outlines are shown as white dashed lines. Scale bars: 1 µm.

G) Overexpression of Pex3 leads to an accumulation of peroxisomes. Quantification of the amount of peroxisomal signal (observed with BFP-SKL) in each peroxisomal cluster in control cells and cells overexpressing Pex3 (*TEF1*pr-*PEX3*). Four independent experiments were performed and 30 cells were analyzed for each experiment. Each small diamond represents a single peroxisomal cluster, and the bigger ones represent the average of each of four independent experiments. Strains were compared using the means of each experiment, with an unpaired, two-tailed Student´s t-test. *** $P < 0.001$.
