# Supplementary material for: Pex3 promotes formation of peroxisome-peroxisome and peroxisome-lipid droplet contact sites
Source: Sci Rep. 2025 Jul 8;15:24480. doi: 10.1038/s41598-025-07934-2 (PMC12238565; doi:10.1038/s41598-025-07934-2)
Supplement: Supplementary file 2 — Supplementary Information 2. [file 41598_2025_7934_MOESM2_ESM.pdf]

Amado et al 2025 - Supplementary Figure 2

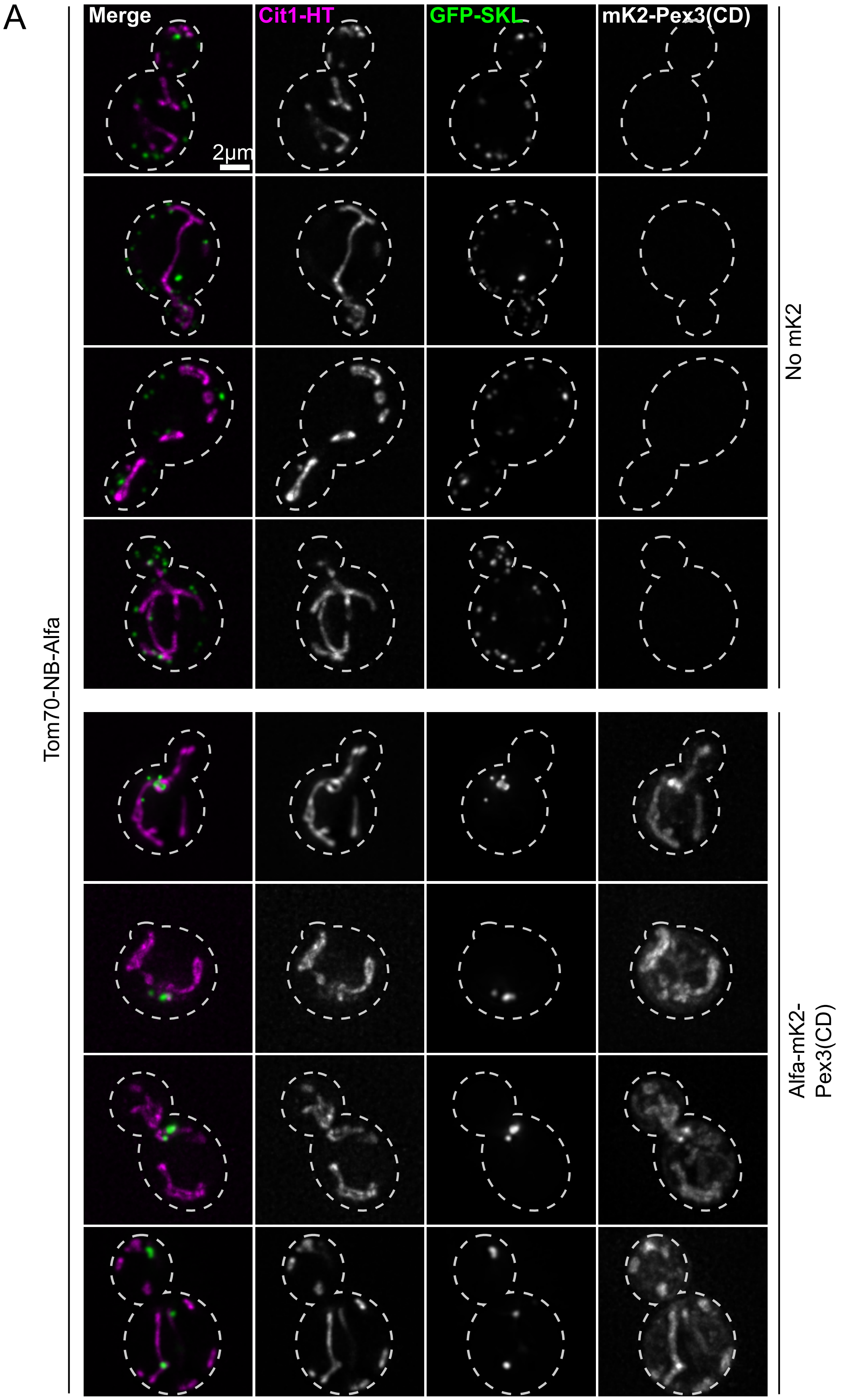

Supplemental Figure 2: The cytosolic domain of Pex3 is able to tether peroxisomes to other organelles.

A) Targeting the cytosolic domain of Pex3 to mitochondria tethers peroxisomes to this organelle. Panel A shows four representative images of the localization of peroxisomes (GFP-SKL) and mitochondria (Cit1-HaloTag) in the presence or absence of AlfaTag -Pex3(CD), with Tom70 fused to Alfa Nanobody in the background. A projection of the maximum intensity of entire Z-stacks is shown for each strain. Cell outlines are shown as white dashed lines. Scale bar: 2 µm.
