# Supplementary material for: Pex3 promotes formation of peroxisome-peroxisome and peroxisome-lipid droplet contact sites
Source: Sci Rep. 2025 Jul 8;15:24480. doi: 10.1038/s41598-025-07934-2 (PMC12238565; doi:10.1038/s41598-025-07934-2)
Supplement: Supplementary file 3 — Supplementary Information 3. [file 41598_2025_7934_MOESM3_ESM.pdf]

Supplemental Figure 3

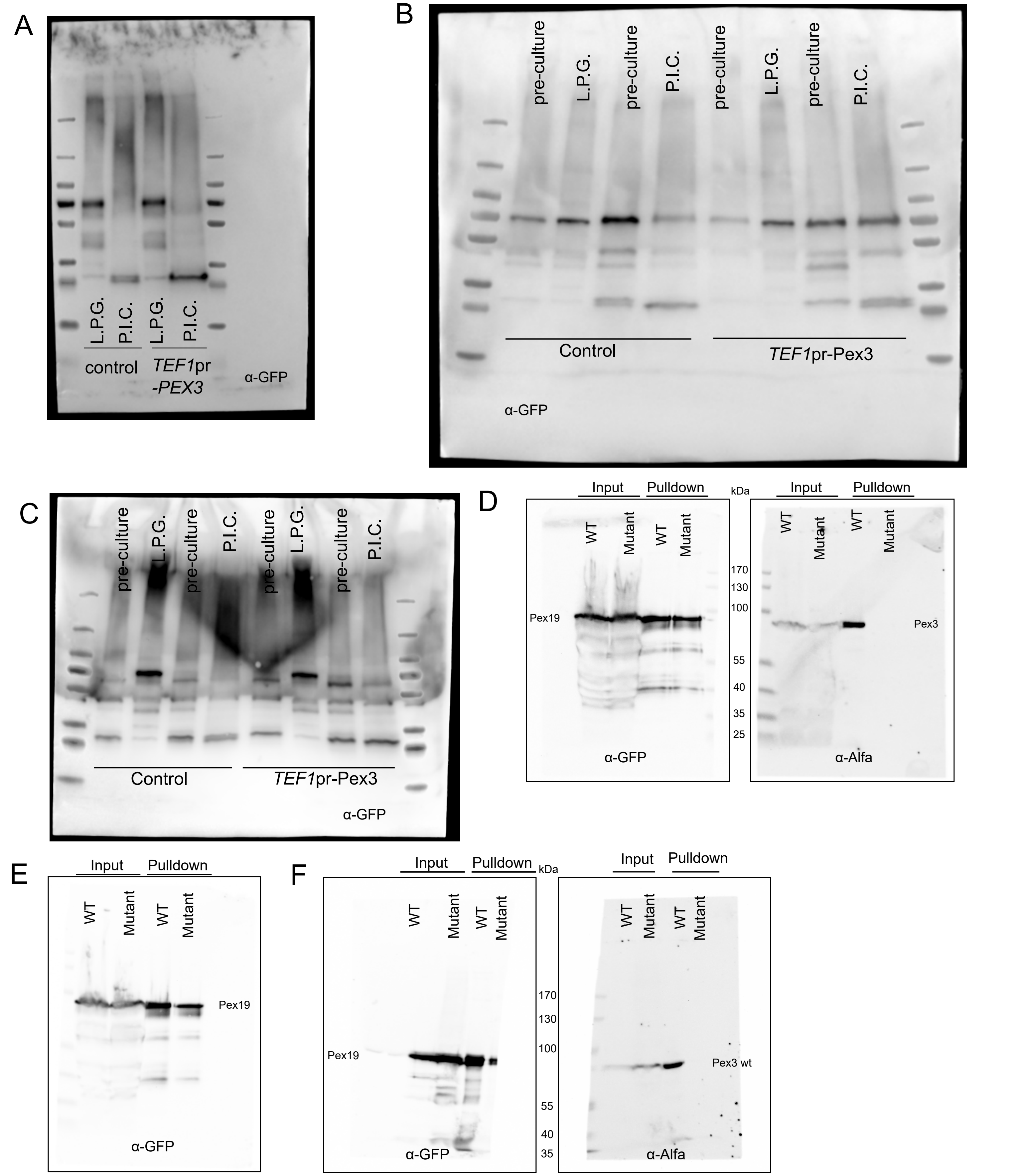

Supplemental Figure 3: Whole western blot membranes of experiments from Figures 4 H and J and additional repetitions

A) The whole Western blot membrane from the experiment in Figure 4H was developed with anti-GFP antibodies.

B and C) Two additional repetitions of the same experiment as Figure 4H and Supplemental Figure 3A. In these two experiments, the pre-cultures were additionally included in the SDSPAGE and Western blot. -

D) Whole Western blot membranes from affinity purification of msGFP2-Pex19 (Figure 4J). The left side of the membrane was developed GFP and the right side against AlfaTag. Pex3 - mKate2-AlfaTag co-purifies with msGFP2-Pex19 while Pex3(W128K L131K)-mKate2-AlfaTag does not.

E) The membrane shown on the right in panel B, previously developed against AlfaTag, was stripped and incubated with anti-GFP antibodies, showing comparable amounts of msGFP2-Pex19 purified for both strains.

D) Whole Western blot membrane of an additional repetition of the experiment shown in Figure 4J and Supplemental Figure 3 D and E
