# Supplementary material for: Pex3 promotes formation of peroxisome-peroxisome and peroxisome-lipid droplet contact sites
Source: Sci Rep. 2025 Jul 8;15:24480. doi: 10.1038/s41598-025-07934-2 (PMC12238565; doi:10.1038/s41598-025-07934-2)
Supplement: Supplementary file 4 — Supplementary Information 4. [file 41598_2025_7934_MOESM4_ESM.pdf]

Amado et al 2025 - Supplementary Figure 4

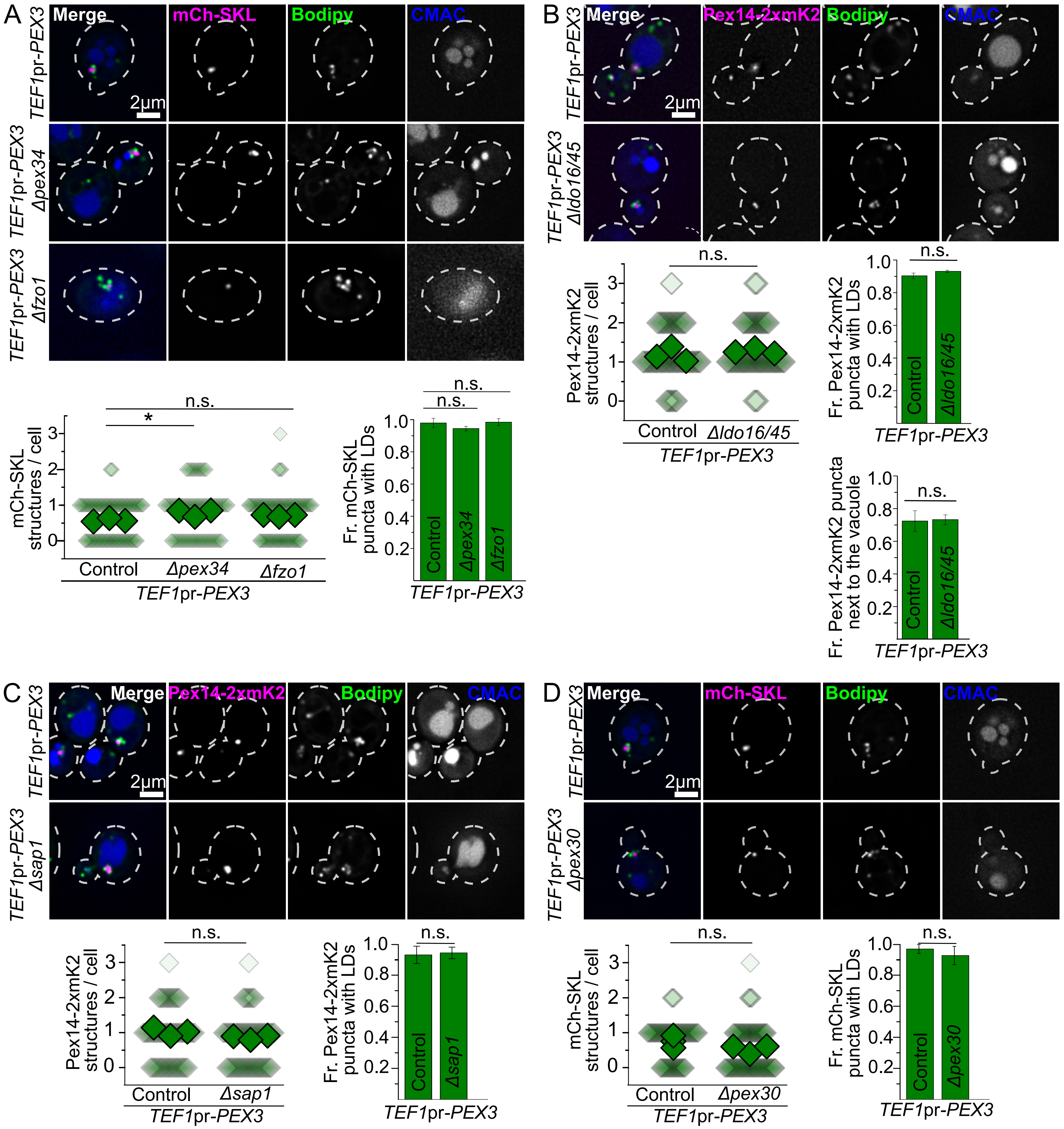

Supplemental Figure 4: Formation of the structure is independent of known peroxisome or lipid droplet contact site tethers.

A) Representative images of strains overexpressing Pex3 (*TEF1*pr-*PEX3*) in control cells and strains lacking Pex35 or Fzo1. Strains express the mCherry-SKL construct to visualize the lumen of the peroxisomes, lipid droplets were stained with Bodipy and the vacuolar lumen was stained with CMAC. Cell outlines are shown as white dashed lines. Scale bars: 2 µm. Quantifications are shown below. For the quantification of the amount of peroxisomal structures per cell, small diamonds correspond to individual cells, bigger diamonds correspond to the average of independent experiments. Three independent experiments were performed and 30 cells were analyzed for each experiment and condition. The strains were compared with ANOVA and a post-hoc Tukey test. n.s., not significant; * $P < 0.05$.

B) Representative images of strains overexpressing Pex3 (*TEF1*pr-*PEX3*) in control cells and strains lacking Ldo16 and Ldo45. Pex14 is tagged with 2xmKate2 to visualize construct to visualize the peroxisomes, lipid droplets were stained with Bodipy and the vacuolar lumen was stained with CMAC. Cell outlines are shown as white dashed lines. Scale bars: 2 µm. Quantifications are shown below. For the quantification of the amount of peroxisomal structures per cell, small diamonds correspond to individual cells, bigger diamonds correspond to the average of independent experiments. Three independent experiments were performed and 30 cells were analyzed for each experiment and condition. The strains compared using an unpaired two-tailed Student's t-test. n.s., not significant.

C) Representative images of a strain overexpressing Pex3 (*TEF1*pr-*PEX3*) and lacking Sap1. Pex14 is tagged to visualize construct to visualize the peroxisomes, lipid droplets were stained with Bodipy and the vacuolar lumen was stained with CMAC. Cell outlines are shown as white dashed lines. Scale bars: 2 µm. Quantifications are shown below. For the quantification of the amount of peroxisomal structures per cell, small diamonds correspond to individual cells, bigger diamonds correspond to the average of independent experiments. Three independent experiments were performed and 30 cells were analyzed for each experiment and condition. The strains were compared using an unpaired two-tailed Student's t-test. n.s., not significant.

D) Representative images of a strain overexpressing Pex3 (*TEF1*pr-*PEX3*) and lacking Pex30. Strains express the mCherry-SKL construct to visualize the lumen of the peroxisomes, lipid droplets were stained with Bodipy and the vacuolar lumen was stained with CMAC. Cell outlines are shown as white dashed lines. Scale bars: 2 µm. Quantifications are shown below. For the quantification of the amount of peroxisomal structures per cell, small diamonds correspond to individual cells, bigger diamonds correspond to the average of independent experiments. Three independent experiments were performed and 30 cells were analyzed for each experiment and condition. The strains were compared using an unpaired two-tailed Student's t-test. n.s., not significant.
