# Supplementary material for: Pex3 promotes formation of peroxisome-peroxisome and peroxisome-lipid droplet contact sites
Source: Sci Rep. 2025 Jul 8;15:24480. doi: 10.1038/s41598-025-07934-2 (PMC12238565; doi:10.1038/s41598-025-07934-2)
Supplement: Supplementary file 5 — Supplementary Information 5. [file 41598_2025_7934_MOESM5_ESM.pdf]

Amado et al 2025 - Supplementary Figure 5

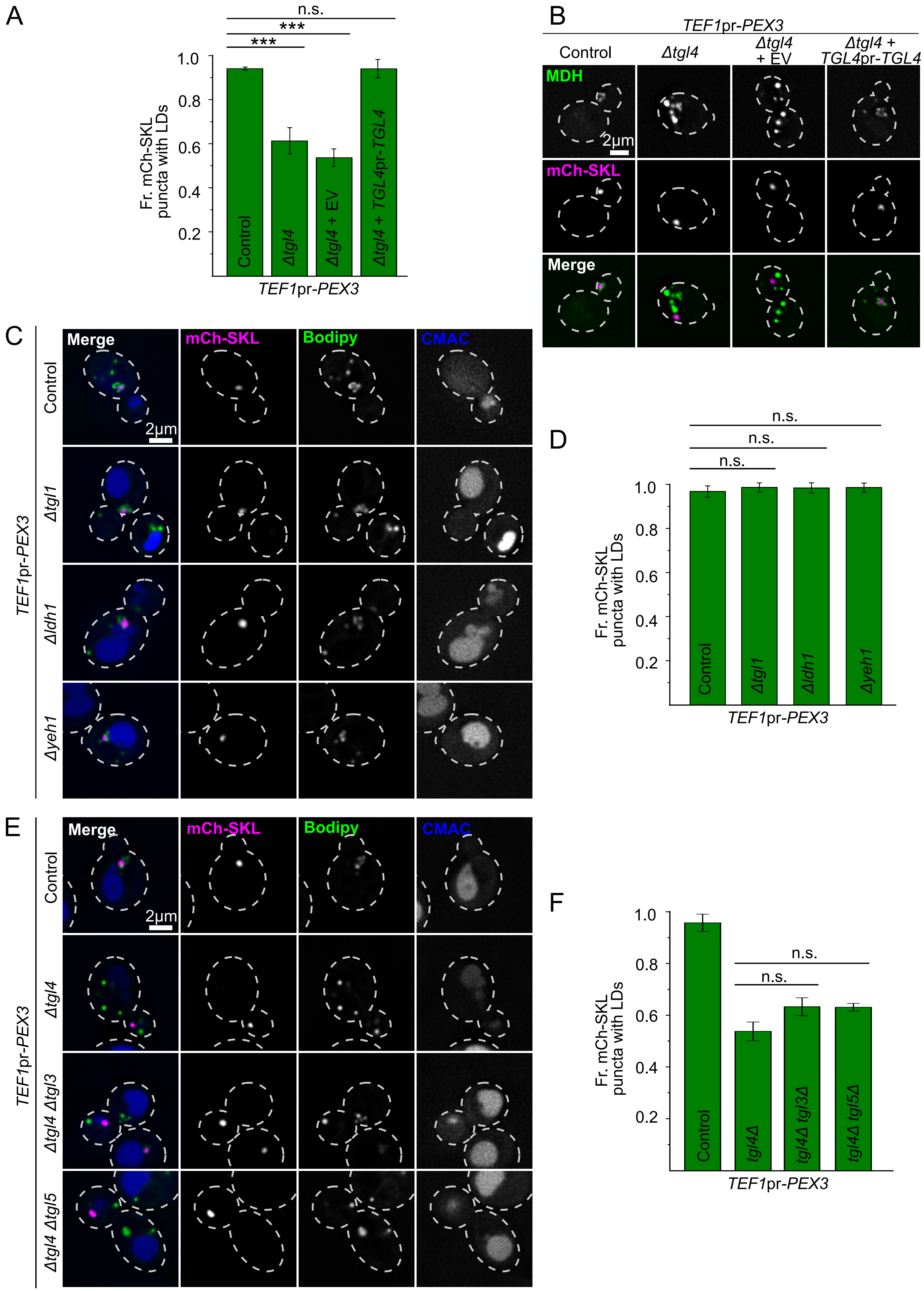

Supplemental Figure 5: Disruption of the lipid droplet association to peroxisomes in Pex3 overexpression cells is specific for Tgl4 among lipases.

A – B) Disruption of peroxisomes-lipid droplets interactions caused by *tgl4Δ* is rescued by re-introducing Tgl4 in a vector. Microscopy experiments were done comparing strains overexpressing Pex3 (TEF1pr-PEX3) in control cells, *tgl4Δ* and in *tgl4Δ* were either an empty vector or a vector containing Tgl4 ORF were introduced. Panel A shows the quantification of the fraction of peroxisomal structures with accumulations of lipid droplets next to them. Three independent experiments were performed and 30 cells were analyzed for each experiment and condition. The different strains were compared by ANOVA and a post-hoc Tukey test. n.s., not significant, *** $P < 0.001$. Panel B shows representative images of the microscopy experiment described above. All strains express mCherry-SKL construct to visualize the lumen of the peroxisomes, lipid droplets were stained with Bodipy and the vacuolar lumen was stained with CMAC. Cell outlines are shown as white dashed lines. Scale bars: 2 μm.

C – D) Deletion of the lipases Tgl1, Ldh1 or Yeh1 has no effect on the phenotype of Pex3 overexpression. Panel C shows representative images of strains overexpressing Pex3 (*TEF1*pr-*PEX3*) in control cells, or *tgl1Δ*, *ldh1* or *yeh1Δ* cells. All strains express mCherry-SKL construct to visualize the lumen of the peroxisomes, lipid droplets were stained with Bodipy and the vacuolar lumen was stained with CMAC. Cell outlines are shown as white dashed lines. Scale bars: 2 μm. Panel D shows the quantification of the fraction of peroxisomal structures with accumulations of lipid droplets next to them in the microscopy experiments described above. Three independent experiments were performed and 30 cells were analyzed for each experiment and condition. The different strains were compared by ANOVA and a post-hoc Tukey test. n.s., not significant.

E – F) Deletion of Tgl5 or Tgl3 in addition to Tgl4 has no additive effect on the Pex3 overexpression phenotype. Panel E shows representative images of strains overexpressing Pex3 (*TEF1*pr-*PEX3*) in control cells, or *tgl4Δ*, *tgl4Δtgl3Δ* or *tgl4Δtgl5Δ* cells. All strains express mCherry-SKL construct to visualize the lumen of the peroxisomes, lipid droplets were stained with Bodipy and the vacuolar lumen was stained with CMAC. Cell outlines are shown as white dashed lines. Scale bars: 2 μm. Panel F shows the quantification of the fraction of peroxisomal structures with accumulations of lipid droplets next to them in the microscopy experiments described above. Three independent experiments were performed and 30 cells were analyzed for each experiment and condition. The different strains were compared by ANOVA and a post-hoc Tukey test. n.s., not significant.
