# Supplementary material for: Pex3 promotes formation of peroxisome-peroxisome and peroxisome-lipid droplet contact sites
Source: Sci Rep. 2025 Jul 8;15:24480. doi: 10.1038/s41598-025-07934-2 (PMC12238565; doi:10.1038/s41598-025-07934-2)
Supplement: Supplementary file 6 — Supplementary Information 6. [file 41598_2025_7934_MOESM6_ESM.pdf]

Amado et al 2025 -Supplemental Figure 6

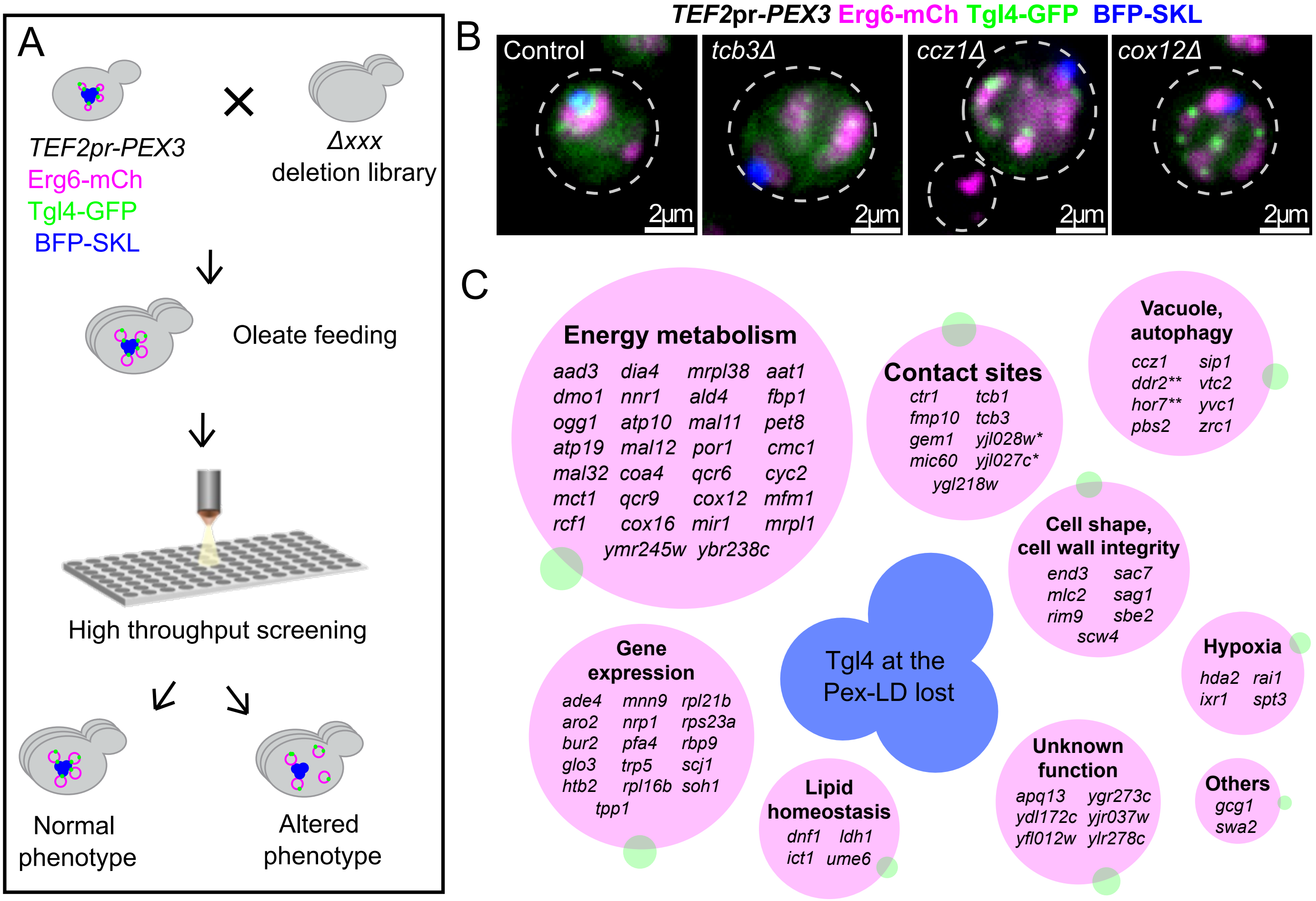

Supplemental Figure 6: A genome-wide screen for mutations affecting the localization of Tgl4 to the peroxisome-lipid droplet interface.

A) A microscopy-based screen for deletions/DAmP alleles that disrupt the enrichment of Tgl4 in the interface between peroxisomes and lipid droplets. Establishment of a deletion library with the overexpression of Pex3 (*TEF2pr-PEX3*), Erg6-mCherry, BFP-SKL and Tgl4-GFP by automated mating and subsequent automated high-content microscopy.

B) Example microscopy images of three deletions (*tcb3Δ, ccz1Δ and cox12Δ*) among the 86 deletions that fully or partially disrupt the enrichment of Tgl4 to the peroxisome -lipid droplet interface. Scale bars: 2 μm.

C) Aggrupation of the hits of the screen into functional categories based on their annotations in the *Saccharomyces* genome database. *, adjacent genes; **, paralogs.
