# Supplementary material for: Pex3 promotes formation of peroxisome-peroxisome and peroxisome-lipid droplet contact sites
Source: Sci Rep. 2025 Jul 8;15:24480. doi: 10.1038/s41598-025-07934-2 (PMC12238565; doi:10.1038/s41598-025-07934-2)
Supplement: Supplementary file 9 — Supplementary Information 7. [file 41598_2025_7934_MOESM9_ESM.docx]

**Supplemental Table 1: *Saccharomyces cerevisiae* strains used in this study**

| **Name** | **Identifier** | **Genotype** | **Source** |
| --- | --- | --- | --- |
| BY4741 | AGMY002 | *MATa his3∆1 leu2∆0 met15∆0 ura3∆0* | Euroscarf |
| W303 | AGMY064 | *matAlpha ura3-52 trp1Δ 2 leu2-3112 his3-11 ade2-1 can1-100* | Ungermann Lab |
| TEF1pr-Pex3 | AGMY192 | *BY4741 – PEX3pr::TEF1pr-NatNT2* | This study |
| mCherry-SKL | AGMY1267 | *BY4741 – LEU2::mCherry-SKL-LEU2* | This study |
| TEF1pr-Pex3 mCherry-SKL | AGMY1268 | *BY4741 – PEX3pr::TEF1pr-NatNT2 LEU2::mCherry-SKL-LEU2* | This study |
| TEF1pr-Pex3-TurboID-V5 | AGMY575 | *BY4741 – PEX3pr::TEF1pr-NatNT2 PEX3::TurboID-V5-KanMX* | This study |
| ΔPex3 | AGMY1847 | *BY4741 – Pex3Δ::HphNT1* | This study |
| ΔPex11 | AGMY894 | *BY4741 – Pex11Δ::KanMX* | This study |
| ΔPex11 TEF1pr-Pex3-msGFP Erg6-2xmKate2 pRS403 TEF1pr-BFP-SKL | AGMY1809 | *BY4741 – Pex11Δ::KanMX PEX3pr::TEF1pr-NatNT2 ERG6::2xmKate2-URA3 HIS3::pRS403-TEF1pr-BFP-SKL-HIS3 PEX3::msGFP-HphNT1* | This study |
| ΔPex11 TEF1pr-Pex3 Erg6-2xmKate2 pRS403 TEFpr-BFP-SKL Pex13-msGFP | AGMY1824 | *BY4741 – Pex11Δ::KanMX PEX3pr::TEF1pr-NatNT2 ERG6::2xmKate2-URA3 HIS3::pRS403-TEF1pr-BFP-SKL-HIS3 PEX13::msGFP-HphNT1* | This study |
| ΔPex11 TEF1pr-Pex3 Erg6-2xmKate2 pRS403 TEFpr-BFP-SKL Pex14-msGFP | AGMY1779 | *BY4741 – Pex11Δ::KanMX PEX3pr::TEF1pr-NatNT2 ERG6::2xmKate2-URA3 HIS3::pRS403-TEF1pr-BFP-SKL-HIS3 PEX14::msGFP-HphNT1* | This study |
| TEF1pr-Pex3 Pex14-2xmKate2 | AGMY1258 | *BY4741 – PEX3pr::TEF1pr-NatNT2 PEX14::2xmKate2-HphNT1* | This study |
| TEF1pr-Pex3 Pex14-2xmKate2 | AGMY1303 | *W303 – PEX3pr::TEF1pr-NatNT2 PEX14::2xmKate2-URA3* | This study |
| ΔLDs | FFY30 | *W303 - Dga1::KanMX4 Llro1::TRP1 Are1::HIS3 Are2::LEU2 ADE2* | Froelich Lab |
| ΔLDs TEF1pr-Pex3 Pex14-2xmKate2 | AGMY1517 | *W303 - PEX3pr::TEF1pr-NatNT2 PEX14::2xmKate2-URA3* | This study |
| TEF1pr-Pex3 ΔPex35 Pex14-2xmKate2 | AGMY1466 | *BY4741 – PEX3pr::TEF1pr-NatNT2 Pex35Δ::HphNT1 PEX14::2xmKate2-URA3* | This study |
| TEF1pr-Pex3 ΔSap1 mCherry-SKL | AGMY1269 | *BY4741 – PEX3pr::TEF1pr-NatNT2 Sap1Δ::HphNT1 LEU2::mCherry-SKL-LEU2* | This study |
| TEF1pr-Pex3 ΔLdo45/16 Pex14-2xmKate2 | AGMY1467 | *BY4741 – PEX3pr::TEF1pr-NatNT2 Ldo45/16Δ::HphNT1 PEX14::2xmKate2-URA3* | This study |
| TEF1pr-Pex3 mCherry-SKL ∆Pex30 | AGMY1929 | *BY4741 – PEX3pr::TEF1pr-NatNT2 LEU2::mCherry-SKL-LEU2 Pex30∆::HphNT1* | This study |
| TEF1pr-Pex3 mCherry-SKL ∆Pex34 | AGMY1933 | *BY4741 – PEX3pr::TEF1pr-NatNT2 LEU2::mCherry-SKL-LEU2 Pex34∆::HphNT1* | This study |
| TEF1pr-Pex3 mCherry-SKL ∆Fzo1 | AGMY1928 | *BY4741 – PEX3pr::TEF1pr-NatNT2 LEU2::mCherry-SKL-LEU2 Fzo1∆::HphNT1* | This study |
| Erg6-2xmKate2 Pex14-HaloTag pRS403 TEF-GFP-Pex3(CD) | AGMY1615 | *BY4741 – ERG6::2xmKate2-URA3 PEX14::HaloTag-MET17 HIS3::pRS403-TEF1pr-GFP-PEX3(CD)-HIS3* | This study |
| Tom70-NbALFA Cit1-Halo GFP-SKL pRS403 TEF1pr-ALFATag-mKate2-Pex3(CD) | AGMY1851 | *BY4741 – TOM70::NbALFA-KanMX CIT1-HaloTag-MET17 LEU2::GFP-SKL-LEU2 HIS3:: pRS403-TEF1pr-ALFATag-mKate2-PEX3(CD)-HIS3* | This study |
| TEF1pr-Pex3 Pex14-2xmKate2 ∆Inp1 | AGMY1536 | *BY4741 – PEX3pr::TEF1pr-NatNT2 PEX14::2xmKate2-HphNT1 Inp1∆::KanMX* | This study |
| TEF1pr-Pex3 Pex14-2xmKate2 ∆Atg36 | AGMY1582 | *BY4741 – PEX3pr::TEF1pr-NatNT2 PEX14::2xmKate2-HphNT1 Atg36∆::KanMX* | This study |
| ΔPex3 pRS403 TEF1pr-Pex3(WT)-mKate2 BFP-SKL | AGMY1896 | *BY4741 – Pex3Δ::HphNT1 HIS3::pRS403-TEF1pr-PEX3(WT)-mKate2-HIS3 LEU2::BFP-SKL-LEU2* | This study |
| ΔPex3 pRS403 TEF1pr-Pex3(W128K, L131K)-mKate2 BFP-SKL | AGMY1897 | *BY4741 – Pex3Δ::HphNT1 HIS3::pRS403-TEF1pr-PEX3(W128K, L131K)-mKate2-HIS3 LEU2::BFP-SKL-LEU2* | This study |
| TEF1pr-msGFP2-Pex19 pRS403 TEF1pr-Pex3(WT)-mKate2-ALFATag | AGMY1919 | *BY4741 – PEX19pr::TEF1pr-msGFP2-NatNT1 HIS3::pRS403-TEF1pr-PEX3(WT)-mKate2-ALFATag-HIS3* | This study |
| TEF1pr-msGFP2-Pex19 pRS403 TEF1pr-Pex3(W128K, L131K)-mKate2-ALFATag | AGMY1920 | *BY4741 – PEX19pr::TEF1pr-msGFP2-NatNT1 HIS3::pRS403-TEF1pr-PEX3(W128K, L131K)-mKate2-ALFATag-HIS3* | This study |
| pRS403 TEF1pr-Pex3(WT)-mKate2 | AGMY1820 | *BY4741 – HIS3::pRS403-TEF1pr-PEX3(WT)-mKate2* | This study |
| pRS403 TEF1pr-Pex3(W128K, L131K)-mKate2 | AGMY1821 | *BY4741 – HIS3::pRS403-TEF1pr-PEX3(W128K, L131K)-mKate2* | This study |
| TEF1pr-Pex3 mCherry-SKL ∆Tgl4 | AGMY1729 | *BY4741 – PEX3pr::TEF1pr-NatNT2 LEU2::mCherry-SKL-LEU2 Tgl4∆::HphNT1* | This study |
| TEF1pr-Pex3 mCherry-SKL ∆Tgl3 | AGMY1844 | *BY4741 – PEX3pr::TEF1pr-NatNT2 LEU2::mCherry-SKL-LEU2 Tgl3∆::KanMX* | This study |
| TEF1pr-Pex3 mCherry-SKL ∆Tgl5 | AGMY1810 | *BY4741 – PEX3pr::TEF1pr-NatNT2 LEU2::mCherry-SKL-LEU2 Tgl5∆::KanMX* | This study |
| TEF1pr-Pex3 mCherry-SKL ∆Tgl1 | AGMY1926 | *BY4741 – PEX3pr::TEF1pr-NatNT2 LEU2::mCherry-SKL-LEU2 Tgl1∆::HphNT1* | This study |
| TEF1pr-Pex3 mCherry-SKL ∆Ldh1 | AGMY1927 | *BY4741 – PEX3pr::TEF1pr-NatNT2 LEU2::mCherry-SKL-LEU2 Ldh1∆::HphNT1* | This study |
| TEF1pr-Pex3 mCherry-SKL ∆Yeh1 | AGMY1936 | *BY4741 – PEX3pr::TEF1pr-NatNT2 LEU2::mCherry-SKL-LEU2 Yeh1∆::HphNT1* | This study |
| ΔPex11 TEF1pr-Pex3 Erg6-2xmKate2 pRS403 TEF1pr-BFP-SKL Tgl4-msGFP | AGMY1782 | *BY4741 – pex11Δ::KanMX PEX3pr::TEF1pr-NatNT2 ERG6::2xmKate2-URA3 HIS3::pRS403-TEF1pr-BFP-SKL-HIS3 TGL4::msGFP-HphNT1* | This study |
| ΔPex11 TEF1pr-Pex3 Erg6-2xmKate2 pRS403 TEF1pr-BFP-SKL Tgl3-msGFP | AGMY1823 | *BY4741 – pex11Δ::KanMX PEX3pr::TEF1pr-NatNT2 ERG6::2xmKate2-URA3 HIS3::pRS403-TEF1pr-BFP-SKL-HIS3 TGL3::msGFP-HphNT1* | This study |
| ΔPex11 TEF1pr-Pex3 Erg6-2xmKate2 pRS403 TEF1pr-BFP-SKL Tgl5-msGFP | AGMY1780 | *BY4741 – pex11Δ::KanMX PEX3pr::TEF1pr-NatNT2 ERG6::2xmKate2-URA3 HIS3::pRS403-TEF1pr-BFP-SKL-HIS3 TGL5::msGFP-HphNT1* | This study |
| Tgl4 (S315G) | AGMY1888 | *BY4741 – TGL4 (S315G)* | This study |
| Tgl4 (S315G) mCherry-SKL TEF1pr-Pex3 | AGMY1895 | *BY4741 – TGL4 (S315G) LEU2::mCherry-SKL-LEU2* | This study |
| TEF2pr-Pex3 Erg6-mCherry BFP-SKL Tgl4-GFP | YMB1326 | *his3∆1, leu2∆0, lys2+/lys+, met15∆0, ura3∆0::BFP-SKL HIS3, can1∆::STE2pr-sp HIS5,  lyp1∆::STE3pr-LEU2, Nat pTEF2-PEX3, TGL4-GFP URA3, ERG6-mCherry HYG* | This study |

**Supplemental Table 2: Plasmids used in this study**

| **Name** | **Identifier** | **Description** | **Yeast selection marker** | **Source** |
| --- | --- | --- | --- | --- |
| pRS403 TEF1pr-BFP-SKL | pAGM197 | Plasmid to be integrated in the *HIS3* locus. It expresses BFP with a Pts1 signal to target it to the lumen of peroxisomes. | HIS3MX6 | This study |
| pRS403 TEF1pr-GFP-Pex3(CD) | pAGM134 | Plasmid to express Pex3 (CD) with an N-terminal GFP tag. | HIS3MX6 | This study |
| pRS403 TEF1pr-AlfaTag-mKate2-Pex3(CD) | pAGM212 | Plasmid to express Pex3 (CD) with an N-terminal ALFA and mKate2 tag. | HIS3MX6 | This study |
| pRS403 TEF1pr-Pex3-mKate2 | pAGM210 | Plasmid to express the full-length Pex3 under the control of the *TEF1* promoter, with a C-terminal mKate2 tag. | HIS3MX6 | This study |
| pRS403 TEF1pr-Pex3 (W128K, L131K)-mKate2 | pAGM211 | Plasmid to express the mutant variant of Pex3 (which does not interact with Pex19), under the control of the *TEF1* promoter, with a C-terminal mKate2 tag. | HIS3MX6 | This study |
| pRS403 TEF1pr-Pex3-mKate2-Alfatag | pAGM225 | Plasmid to express the full-length Pex3 under the control of the *TEF1* promoter, with a C-terminal mKate2 and ALFA tag. | HIS3MX6 | This study |
| pRS403 TEF1pr-Pex3 (W128K, L131K)-mKate2-Alfatag | pAGM226 | Plasmid to express Pex3(W128K, L131K) under the control of the TEF1 promoter with a C-terminal tag consisting of mKate2 and ALFA tag. | HIS3MX6 | This study |
| pRS315 mCherry-SKL | CU5065 | Plasmid to express mCherry with a Pts1 signal to target it to the lumen of peroxisomes. | LEU2 | Gift from Judith Müller (Institute of Molecular Genetics and Cell Biology, Ulm University) |
| pRS415 GFP-SKL | CU5066 | Plasmid to express GFP with a Pts1 signal to target it to the lumen of peroxisomes. | LEU2 | (Schäfer et al., 2004) |
| pRS415 TEF1pr-BFP-SKL | pAGM224 | Plasmid to express BFP with a Pts1 signal to target it to the lumen of peroxisomes. | LEU2 | This study |
| pRS403 TGL4pr-Tgl4-msGFP2 | pAGM242 | Plasmid to express Tgl4 under its endogenous promoter, with a C-terminal msGFP2 tag. | HIS3MX6 | This study |

**Supplemental Table 3: Oligonucleotides used in this study**

| **Identifier** | **Sequence** |
| --- | --- |
| oAGM090 | CCTTATCACGTTGAGCCATTAGTATCAATTTGCTTACCTGTATTCCTTTACATCCTCCGCGTATATAGTTTC  GTCTACCC |
| oAGM091 | GGGTAGACGAAACTATATACGCGGAGGATGTAAAGGAATACAGGTAAGCAAATTGATACTAATGGCTCAA  CGTGATAAGG |
| oAGM470 | AAAGCGGCCGCCAAGAGATGGTTGTATAAACA |
| oAGM471 | AAACCGCGGTTAAGGCTTGAAGGAAAACG |
| oAGM748 | CTAGCAAACTGGGGCACAGAAGATCCACTAGTTCTAGAGC |
| oAGM749 | ACGTAATTCCTCTTCCAACCTGGAGGGCATGGATCCCTTAGATTAGATTG |
| oAGM750 | GAAGAGGAATTACGTCGTCGTTTGACCGAAGTGAGCGAGCTGATTAAGGA |
| oAGM751 | GCTCTAGAACTAGTGGATCTTCTGTGCCCCAGTTTGCTAG |
| oAGM831 | GTTGAAGAACGAGAAGGAACTAAAGAGTTTGATCAAGCTGGTG |
| oAGM832 | GTTCCTTCTCGTTCTTCAACTCAGCTTTACTTTTTAACGGCG |
| oAGM839 | AAGCATAGCAATCTAATCTAAGTTTTCTAGATGTCCCCGGGTACCAGATCTATGAGTA |
| oAGM840 | CGACGGTATCGATAAGCTTGATATCGAATTTTAGAGTTTGCTTTTGTATAGTTCATCCATGCCATGT |
| oAGM841 | ACATGGCATGGATGAACTATACAAAAGCAAACTCTAAAATTCGATATCAAGCTTATCGATACCGTCG |
| oAGM842 | TACTCATAGATCTGGTACCCGGGGACATCTAGAAAACTTAGATTAGATTGCTATGCTT |
| oAGM851 | GCAATCTAATCTAAGGGATCCATGGCCCCAAATC |
| oAGM852 | CAGATGAAGATCTAACCGCGGTGGAGCTCC |
| oAGM853 | TAAGGGATCCATGGCCCCAAATCAAAGATC |
| oAGM854 | CTGCAGCGTACGAGGCTTGAAGGAAAACGAGC |
| oAGM855 | TCCTTCAAGCCTCGTACGCTGCAGGTCGACGG |
| oAGM856 | CACCGCGGTTAGATCTTCATCTGTGCCCC |
| oAGM873 | GTGATTAGTGGTAGCAGTGCGTTTTAGAGCTAGAAATAGCAAGTTAAAATAAGG |
| oAGM874 | GCACTGCTACCACTAATCACGATCATTTATCTTTCACTGCGGAG |
| oAGM875 | TTGAATTGGATTTATTACCCAGAGTGATTAGTGGTAGCGGTGCTGGTGCAATTGTAGCAAGCATATTATCT  GTCCATCA |
| oAGM910 | TCTAGATTGGAAGAAGAATTGAGAAGAAGATTGACTGAATGAAGATCTAACCGCGGTGG |
| oAGM911 | TTCAGTCAATCTTCTTCTCAATTCTTCTTCCAATCTAGATCTGTGCCCCAGTTTGCTAG |
| oAGM930 | AAAGTCGACTCAGACTTTTATTACATATACC |
| oAGM1004 | GGATCCACCGGTCGCCACCATGGTGAGCAAGGGCGAGGA |
| oAGM1005 | GATCTAGAGTCGCGGCCGCTTTACTTGTACAGCTCGTCC |
| oAGM1006 | GGACGAGCTGTACAAGTAAAGCGGCCGCGACTCTAGATC |
| oAGM1007 | TCCTCGCCCTTGCTCACCATGGTGGCGACCGGTGGATCC |

**Supplemental Table 4: Antibodies used in this study**

| **Reagent or Resource** | **Source** | **Identifier** |
| --- | --- | --- |
| Rabbit polyclonal anti-Alfa | NanoTag Biotechnologies | Cat# 1581  RRID: AB_3075997 |
| Rabbit monoclonal anti-GFP | Roche | Cat# 11814460001  RRID: AB_390913 |
| DyLight 800 Goat anti Rabbit IgG | Thermo Scientific | Cat# SA5-35571;  RRID: AB_614947 |
| Goat IgG anti-rabbit HRP | Dianova | Cat#: 111-035-003  RRID:AB_2313567 |
| Rabbit GFP tag polyclonal | proteintech | Cat#: 50430-2-AP  RRID:AB_11042881 |

**Supplemental Table 5: Hits from genome-wide screen for deletions that disrupt Tgl4 localization at lipid droplet-peroxisome contact site**

| **Systematic name** | **Standard name** |  | **Systematic name** | **Standard name** |
| --- | --- | --- | --- | --- |
| YOR037W | CYC2 |  | YGR279C | SCW4 |
| YOR374W | ALD4 |  | YLR038C | COX12 |
| YLR377C | FBP1 |  | YJR120W | DMO1 |
| YGL218W |  |  | YML072C | TCB3 |
| YLR393W | ATP10 |  | YJR077C | MIR1 |
| YLR278C |  |  | YDL172C |  |
| YPL060W | MFM1 |  | YBR131W | CCZ1 |
| YFR033C | QCR6 |  | YJL128C | PBS2 |
| YPR124W | CTR1 |  | YGL127C | SOH1 |
| YAL048C | GEM1 |  | YER182W | FMP10 |
| YML060W | OGG1 |  | YGL148W | ARO2 |
| YML030W | RCF1 |  | YBR299W | MAL32 |
| YMR214W | SCJ1 |  | YCR107W | AAD3 |
| YMR243C | ZRC1 |  | YFL004W | VTC2 |
| YMR245W |  |  | YFL012W |  |
| YMR300C | ADE4 |  | YER163C | GCG1 |
| YOL003C | PFA4 |  | YGR273C |  |
| YOR086C | TCB1 |  | YER166W | DNF1 |
| YDR392W | SPT3 |  | YGR289C | MAL11 |
| YBR204C | LDH1 |  | YGR292W | MAL12 |
| YDR116C | MRPL1 |  | YLR226W | BUR2 |
| YBR238C |  |  | YNL055C | POR1 |
| YGR183C | QCR9 |  | YPL050C | MNN9 |
| YHR011W | DIA4 |  | YBL002W | HTB2 |
| YKL106W | AAT1 |  | YGL070C | RPB9 |
| YLR218C | COA4 |  | YNL069C | RPL16B |
| YOR221C | MCT1 |  | YJL003W | COX16 |
| YKL137W | CMC1 |  | YJL027C |  |
| YGR118W | RPS23A |  | YJL028W |  |
| YKL032C | IXR1 |  | YJR004C | SAG1 |
| YKL170W | MRPL38 |  | YJR037W |  |
| YGL246C | RAI1 |  | YDL167C | NRP1 |
| YDR207C | UME6 |  | YNL003C | PET8 |
| YPL079W | RPL21B |  | YNL084C | END3 |
| YPR188C | MLC2 |  | YER122C | GLO3 |
| YNL200C | NNR1 |  | YJL075C | APQ13 |
| YGL026C | TRP5 |  | YOL052C-A | DDR2 |
| YDR295C | HDA2 |  | YOL077W-A | ATP19 |
| YDR320C | SWA2 |  | YMR156C | TPP1 |
| YKR016W | MIC60 |  | YLR099C | ICT1 |

| **Systematic name** | **Standard name** |
| --- | --- |
| YOR087W | YVC1 |
| YDR389W | SAC7 |
| YDR422C | SIP1 |
| YDR351W | SBE2 |
| YMR063W | RIM9 |
| YMR251W-A | HOR7 |
